# High spatial resolution assessment of air quality in urban centres using lichen carbon, nitrogen and sulfur contents and stable-isotope-ratio signatures

**DOI:** 10.1007/s11356-023-26652-8

**Published:** 2023-03-30

**Authors:** Daniel Niepsch, Leon J. Clarke, Jason Newton, Konstantinos Tzoulas, Gina Cavan

**Affiliations:** 1 Department of Natural Sciences, Faculty of Science and Engineering, Manchester Metropolitan University, Manchester, M1 5GD UK; 2 Stable Isotope Ecology Laboratory, Scottish Universities Environmental Research Centre (SUERC), East Kilbride, G75 0QF UK

**Keywords:** Urban environment, Lichen biomonitoring, Source apportionment, Manchester (UK), *Xanthoria parietina*, *Physcia* spp

## Abstract

Air pollution and poor air quality is impacting human health globally and is a major cause of respiratory and cardiovascular disease and damage to human organ systems. Automated air quality monitoring stations continuously record airborne pollutant concentrations, but are restricted in number, costly to maintain and cannot document all spatial variability of airborne pollutants. Biomonitors, such as lichens, are commonly used as an inexpensive alternative to assess the degree of pollution and monitor air quality. However, only a few studies combined lichen carbon, nitrogen and sulfur contents, with their stable-isotope-ratio signatures (δ^13^C, δ^15^N and δ^34^S values) to assess spatial variability of air quality and to ‘fingerprint’ potential pollution sources. In this study, a high-spatial resolution lichen biomonitoring approach (using *Xanthoria parietina* and P*hyscia* spp.) was applied to the City of Manchester (UK), the centre of the urban conurbation Greater Manchester, including considerations of its urban characteristics (e.g., building heights and traffic statistics), to investigate finer spatial detail urban air quality. Lichen wt% N and δ^15^N signatures, combined with lichen nitrate (NO_3_^−^) and ammonium (NH_4_^+^) concentrations, suggest a complex mixture of airborne NO_x_ and NH_x_ compounds across Manchester. In contrast, lichen S wt%, combined with δ^34^S strongly suggest anthropogenic sulfur sources, whereas C wt% and δ^13^C signatures were not considered reliable indicators of atmospheric carbon emissions. Manchester’s urban attributes were found to influence lichen pollutant loadings, suggesting deteriorated air quality in proximity to highly trafficked roads and densely built-up areas. Lichen elemental contents and stable-isotope-ratio signatures can be used to identify areas of poor air quality, particularly at locations not covered by automated air quality measurement stations. Therefore, lichen biomonitoring approaches provide a beneficial method to supplement automated monitoring stations and also to assess finer spatial variability of urban air quality.

**Graphical abstract:**

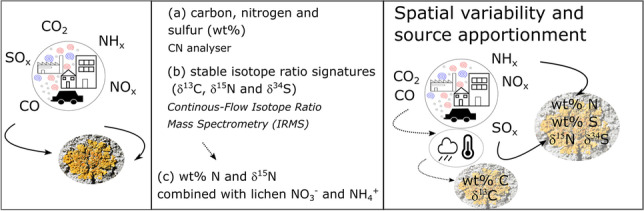

**Supplementary Information:**

The online version contains supplementary material available at 10.1007/s11356-023-26652-8.

## Introduction

Urban air pollution and poor air quality have become an increasing worldwide concern, with urban populations increasingly exposed to a large number of airborne pollutants (Academy of Science of South Africa et al. 2019). Globally, 7 million premature deaths are linked to air pollution each year, with about 40,000 such deaths within the UK (The Royal College of Physicians 2016; WHO 2018). Atmospheric pollutants, such as carbon monoxide/dioxide (CO and CO_2_), sulfur dioxide (SO_2_) and nitrogen oxides (NO_x_; combining nitric oxide (NO) and nitrogen dioxide (NO_2_)) as well as reduced nitrogen (NH_x_), are linked to deleterious human health impacts, including respiratory and cardiovascular disease and damage to the human organ system (Academy of Science of South Africa et al. 2019; Berner and Felix 2020; Gu et al. 2021; Schraufnagel et al. 2019). Negative health impacts of CO include replacement of oxygen in haemoglobin, and damage to the nervous and cardiovascular systems (Kampa and Castanas 2008; NAEI 2018a). Sulfur dioxide (SO_2_) has long been a pollutant of interest due to its role in forming winter-smog and acid rain, with impacts on the human respiratory system; e.g., coughing, nose and throat irritation, bronchoconstriction and dyspnoea (Kampa and Castanas 2008; NAEI 2018b; WHO 2018). Short- or long-term exposure to nitrogen dioxide (NO_2_) can have a variety of health impacts, of which asthma, respiratory disorder, reduced lung function, bronchitis and cancer are of major concern; there also is evidence that high NO_2_ levels can impair neurodevelopment in children (Moldanová et al. 2011; The Royal College of Physicians 2016; WHO 2013). Comparably reduced nitrogen compounds (e.g. nitrate—NO_3_ and ammonia/ammonium – NH_3_/NH_4_) reportedly impact on the cardiovascular and respiratory system (Kim et al. 2012; Son et al. 2012; WHO 2013).

Atmospheric pollution by carbon and sulfur compounds is linked closely to fossil fuel combustion (EPA 2008; WHO 2018). Nitrogen oxides (NO_x_) are released into urban environments from combustion processes, heating, energy production and road traffic (Boltersdorf et al. 2014; DEFRA and DfT 2017). In particular, diesel vehicles are responsible for emissions of primary NO_2_, especially when moving slowly (Abdull et al. 2020). According to EU/UK legislation (Air quality Directive 2008/50/EC; EU 2008) local authorities are obliged to monitor airborne pollutants, e.g., CO, NO_2_ and SO_2_, and comply with set regulatory limit values to limit negative human health impacts (EU 2008). Within the UK, automated air-quality monitoring stations (Automatic Urban and Rural Network – AURN; DEFRA 2020) generate continuous records of air quality. However, these are high-cost infrastructures, restricted in number, only record localised air quality, and are not identifying wider scale areas of poor(er) air quality. Urban environments and associated air quality are complex systems with various influencing factors, including building heights and density, road traffic and localised meteorological conditions, these all influencing spatial variability of pollutant distributions (Salmond and McKendy 2009). This urban environment complexity highlights the necessity to apply additional monitoring methods to achieve finer spatial detail of urban air quality, with implications for understanding deleterious impacts on human health and thereafter informing air quality improvement strategies.

Passive biomonitors are organisms that are already part of a natural ecosystem and active biomonitors are organisms that can be easily transferred into an area under investigation. Both types of biomonitors have been used extensively in environmental pollution studies; e.g., use of tree bark, tree leaves and needles, mosses and lichens (Boltersdorf et al. 2014; Forbes 2015). These natural materials are commonly used where costly technical apparatus cannot be afforded (Forbes et al. 2015). Lichens have been shown to be excellent organisms to monitor atmospheric pollution and air quality, because they can be used to obtain qualitative and quantitative information about environmental conditions (Forbes et al. 2015). Lichens are organisms comprising a self-supporting association between at least a fungal (mycobiont) and a photosynthetic (photobiont) partner, the latter can be either green-algae and/or cyanobacteria (Kirschbaum and Wirth 2010; Nash III 2008; Van der Wat and Forbes 2015). Due to their morphology, lacking roots and cuticle, lichens take up nutrients and airborne pollutants directly from the surrounding atmosphere, by dry and/or wet deposition (Forbes 2015; Forbes et al. 2015).

Only a few previous studies utilised lichen carbon (C) and/or sulfur (S) contents in lichen biomonitoring studies (e.g., Beck and Mayr 2012; Carreras and Pignata 2002; Vingiani et al. 2004). However, numerous studies have used lichens as monitors for nitrogen (N) compounds in (urban) environments, including assessment of the effects of nitrogen pollution on lichen species abundance and distribution patterns (e.g., Bermejo-Orduna et al. 2014; Boltersdorf and Werner 2013, 2014; Gerdol et al. 2014; Pinho et al. 2017). Moreover, lichen nitrogen contents can reflect airborne nitrogen loads, including from anthropogenic impacts (Boltersdorf et al. 2014; Frati et al. 2006; Gadsdon et al. 2010), indicating their suitability for urban air quality studies. In contrast, lichen stable-isotope compositions have not been used to the same extent in air pollution studies, compared to soil, groundwater, precipitation and moss samples (Cape et al. 2004; Widory 2007). A small number of prior studies have investigated the use of lichen stable-isotope-ratio signatures to characterise anthropogenic sources of airborne carbon, nitrogen and sulfur compounds in urban (i.e., Ruhr-Area Germany, Mexico City, Mexico, and Sydney, Australia) and remote/rural (i.e., Antarctic, Germany and China) environments (Batts et al. 2004; Boltersdorf et al. 2014; Boltersdorf and Werner 2013; Lee et al. 2009; López-Veneroni 2009; Wadleigh 2003; Wadleigh and Blake 1999; Wiseman and Wadleigh 2002; Xu et al. 2021).

This study is a first application of a high-spatial resolution lichen passive biomonitoring approach, using lichen species *Xanthoria parietina* and *Physcia* spp. in the City of Manchester (UK). The primary aim was: (i) to assess spatial variability of air quality within a large-scale urban environment, Manchester (UK), using lichen carbon, nitrogen and sulfur contents (wt%), combined with their stable-isotope-ratio signatures (expressed as δ^13^C, δ^15^N and δ^34^S values). The latter were used to: (ii) potentially identify major occurrences, and assess potential sources, of airborne pollution across the centre of Manchester. To further pinpoint potential pollution sources: (iii) lichen wt% N and δ^15^N were combined with previously published lichen nitrate (NO_3_^−^) and ammonium (NH_4_^+^) contents. Lichen samples collected from a rural setting also have been used to compare contrasting environments experiencing different levels and types of airborne pollution.

## Materials and methods

### Study area – the City of Manchester (UK)

The urban conurbation of Greater Manchester, located in northwest England (53.4808°N, 2.2426°W), is the third largest UK urban agglomeration (ONS 2021). The City of Manchester (hereafter Manchester), situated at the centre of this conurbation, covering an area ca. 11,500 hectares, with a population of about 566,000 in 2018 (Manchester City Council 2019). It is located in the northwest of England, where climatic conditions can vary greatly and include the wettest and coldest place in England (Met Office 2015). The annual average temperatures in Greater Manchester for the period 1991–2020 (recorded at Rochdale climate station; Lat.: 53.6, Long: -2.183; Met Office 2022) were 6.09 °C (minimum.) and 13.13 °C (max.), with February being the coldest months (average min. temperature 1.41 °C) and July being the warmest (average max. temperature 20.01 °C). The annual average sunshine (hours) and rainfall (mm) were 1265.48 h and 1197.22 mm, respectively (Met Office 2022). Climatic data for the sampling period of this study, between 2016 and 2018, are displayed in Fig. S1.

**Fig. 1:**
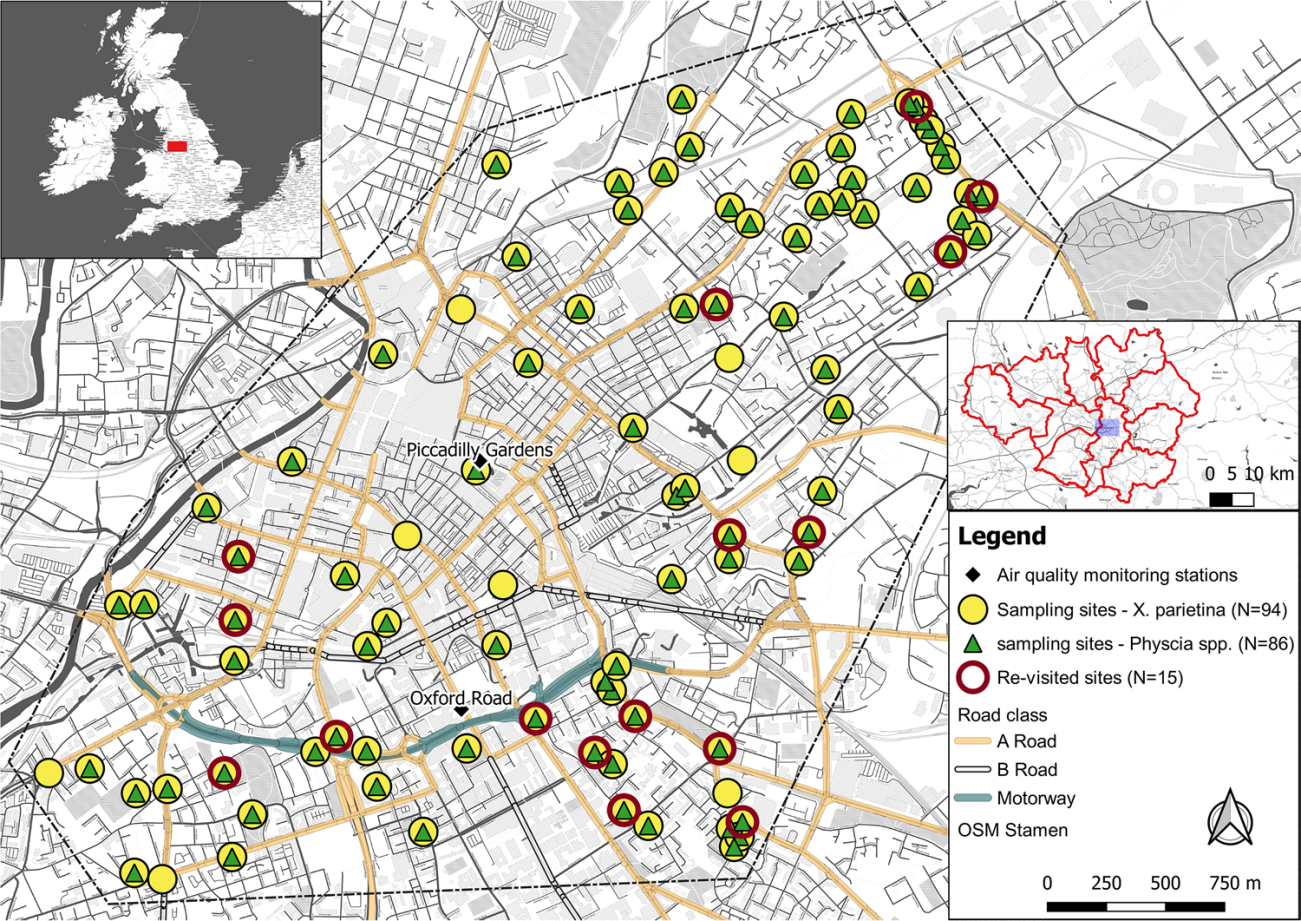
Lichen sampling sites (*X. parietina*: yellow circle; *Physcia* spp.: green triangle; re-sampled sites in 2018: red outline) distributed across Manchester city centre (research area displayed as dotted line; NE-SW transect). Two automated air-quality monitoring stations (labelled black diamonds) and major road classes also are shown

Public health problems in Manchester that are closely linked to poor air quality particularly relate to premature deaths due to cardiovascular diseases, cancer and respiratory diseases, these being the highest in England compared to the English national average (Manchester City Council 2019; Regan 2018). For instance, childhood hospital admissions for asthma (1^st^ rank in England) and emergency Chronic Obstructive Pulmonary Disease (COPD) hospital admissions over twice the UK national rate illustrate major public health issues within Manchester (Regan 2018). According to EU/UK legislation, the City of Manchester is recording atmospheric pollutants within its central parts, using two automated air-quality monitoring stations, situated at Piccadilly Gardens (Latitude: 53.481520, Longitude: -2.237881, urban centre location; Fig. 1) and on Oxford Road (Latitude 53.472077, Longitude -2.239001, urban traffic location; Fig. 1). However, exceedances of the EU/UK NO_2_ regulatory value (annual average of 40 µg m^−3^) are recorded at both monitoring stations, with Oxford Road air quality monitoring station notably exceeding the annual limit value between January 2010 and January 2019 (Fig. S2 a and b). In particular, Manchester Oxford Road is one of the busiest bus routes in Europe and the highest NO_2_ distributions within Manchester are mainly associated with arterial roads leading into the city centre (Manchester City Council 2016; Martin et al. 2011; Regan 2018). Comparably, Niepsch et al. (2021a) reported elevated NO_2_ concentrations (measured by passive diffusion tubes) across Manchester’s city centre and along its major road network. Although providing continuous data, the two automated air quality monitoring stations do not provide information on the wider spatial distribution of airborne pollutants. This key limitation requires alternative measurement options to evaluate air quality in greater spatial detail; i.e., the application of a lichen biomonitoring approach to provide further insights into atmospheric pollution variability across the larger urban environment.

The research area represents a SW–NE transect across central Manchester (Fig. 1) and includes different land-use types; e.g., town centre and retail, residential and industrial areas, as well as open green spaces (parks). The city centre is characterised by high buildings, high density and slow traffic, including public transport (buses, trams and trains) particularly during peak hours (AM and PM peak; Highway Forecasting and Analytical Services 2015). These characteristics suggest vehicular emissions as a major cause of poor air quality and air pollution, which may subsequently impact on the Manchester population health.

### Lichen sampling and preparation

The epiphytic, i.e. growing on plant surfaces, lichen species *Xanthoria parietina* and two *Physcia* spp. (*Physcia adscendens* and *Physcia tenella*) were used in this study; the latter was combined into a single sample (per location), because both look similar, making it difficult to differentiate younger specimens and were both used because they were often found together or are grown into each other; Dobson 2011; Kirschbaum and Wirth 2010). The lichen species used are considered ‘nitrophytes’; being able to tolerate and withstand high concentrations of nitrogenous airborne pollutants. For this reason they are abundant in different environments, e.g. urban and rural areas, making these lichens suitable for large spatial scale air quality biomonitoring studies (Dobson 2011; Kirschbaum and Wirth 2010).

Urban *X. parietina* (N = 94; Fig. 1) and *Physcia* spp. (N = 86; Fig. 1) lichens were sampled from street trees (e.g., *Acer* spp., *Tilia* spp. and *Fraxinus* spp.) situated across Manchester (Fig. 1), between the end of June 2016 and the end of October 2017. Lichen material was obtained from small twigs and branches using a tree pruner (at heights between 2–4 m) from the cardinal direction facing the major road. Vertical NH_3_ profiles vary with height in urban environments (Zhang et al. 2018), which may impact on lichen stable isotope ratio signatures. However, the profile is more pronounced during night-time compared to daytime (Erisman et al. 1988), when lichens were sampled in this study (i.e. during dry days). Further, vertical pollutant gradients were out of scope for this study.

Depending on lichen cover (on twigs and small branches), one or more cardinal direction (clockwise rotation) of a street tree was sampled and merged into a single sample. Twigs and small branches were targeted for collection to facilitate sampling of younger lichen samples for assessment of more recent air quality. It was not possible to sample only one tree species, due to the low tree cover within the city centre (< 10%; City of Trees 2018), tree availability (i.e., estimated 9.7% street trees in city tree cover, Red Rose Forest 2008) and accessibility and the diversity of ornamental and planted (younger) trees across Manchester (City of Trees 2018). However, tree species with similar bark substrate acidity (Kirschbaum and Wirth 2010) were sampled for lichens. It needs to be stated that environmental factors (i.e. light availability and illumination, precipitation, humidity, bark age, corrugation and acidity) are important factors for lichen community development (Broad 1989; Forbes et al. 2015; Shukla et al. 2014). However, less acidic conditions are generally found in the upper, younger parts of a tree (Broad 1989) where the presence of nitrophytic lichen species on twigs and branches (e.g. *X. parietina* and *Physcia* spp.) suggests the presence of atmospheric nitrogen compounds and similar environmental conditions (e.g., eutrophication) across Manchester. Hence, impacting on lichen colonisation and succession favouring the growth of nitrogen-preferring species such as *X. parietina* (Larsen Vilsholm et al. 2009).

Collected twigs with lichens were stored in paper bags and returned to the laboratory; lichen material then was carefully scraped off the bark using a stainless-steel scalpel and non-lichen detritus was removed under an illuminated magnifying glass. Dry lichen material was ground and homogenised into powder using an agate pestle and mortar and stored in glass vials (away from chemicals and in the dark at room temperature [20°]) until chemical analyses. A second phase of urban lichen sampling was undertaken in 2018 (revisiting the same trees; N = 15; red circles in Fig. 1) to assess the scale of any temporal variability of carbon, nitrogen and sulfur (CNS) contents (%wt) in urban lichen samples, since evidence for temporal variability in lichen chemistry will impact the analysis and interpretation of spatial variability of air quality.

To evaluate whether urban lichen %wt CNS and stable-isotope-ratio signatures are diagnostic for urban environments, *X. parietina* lichens also were sampled from around a poultry farm in a rural setting (N = 12; Fig. S3). Rural lichens were sampled in close proximity (between 50 to 500 m) and on a general south-west transect away from the farm (between 1 and 3 km), influenced by site accessibility, i.e. fenced and hedged agricultural fields and private property. These lichens were sampled from oak (*Quercus* spp.) and hawthorn (*Crataegus* spp.) trees, using the same procedure as undertaken in the urban environment. In contrast to the urban environment, *X. parietina* was sampled from trees with potentially different physico-chemical bark properties. However, *Quercus* and *Crataegus* spp. was primarily sampled in close proximity to the poultry farm, being generally completely covered with lichens, suggesting a surplus of atmospheric nitrogen. For instance, ammonia (NH_3_) emissions in rural environments are primarily derived from animal waste and chemical fertiliser, whereas NH_3_ (and NO_x_) in urban environments is predominantly emitted by vehicular sources (Bishop and Stedman 2015; Cape et al. 2004; NAEI 2016; Stritzke et al. 2015; Sun et al. 2017). The lichen *X. parietina* flourishes in such nitrogen-rich habitats, e.g. areas high in NO_x_ and NH_x_ compounds, and is found ubiquitously in urban environments and in proximity to domestic livestock due to increases atmospheric nitrogen compounds (Gaio-Oliveira et al. 2004; Pinho et al. 2009; Sparrius 2007; Van Herk 2003; Van Herk et al. 2003), subsequently allowing for robust comparison of lichen chemistry between rural and urban environments.

### Lichen carbon, nitrogen and sulfur contents

Lichen total carbon and total nitrogen contents, expressed as percentage by weight (wt%), were determined using a Leco TruSpec®’ CN analyser. Lichen sulfur contents (wt% S) were simultaneously determined with stable-isotope-ratios, following the procedure described in “*Lichen stable-isotope-ratio ratios (*δ^*13*^*C,* δ^*15*^*N and* δ^*34*^*S values)*”.

About 0.15 g of each sample was weighed into a tin foil and CN analyser, and calibration was undertaken using the standard Ethylenediaminetetra-acetic acid (LECO®; EDTA 502–092). Rice flour (LECO® reference material; *N* = 32) and Certified Reference Material (CRM) No. 482 (BCR – Trace elements in lichen *Pseudevernia furfuracea*, sample identification No. 594; *N* = 31) were used to quantify accuracy and precision of measurements, as well as assess batch-to-batch variability and any experimental bias between the five different sets of samples. Analytical accuracy (± precision, as coefficient of variation—%CV; Table S1) for the lichen CRM was 102% (± 2.19%CV) for wt% N and 99% (± 1.25%CV) for wt% C (Quevauviller et al. 1996). LECO Rice Flour accuracy (± precision, as %CV) was at 88% (± 3.85%CV) for wt% N and 92% (± 1.00%CV) wt% C. Batch-to-batch corrections were not undertaken, because of good repeatability (< 5%CV) for wt% C and wt% N between each analytical batch. Lichen CRM wt% S (by IRMS; “*Lichen stable-isotope-ratio ratios (*δ^*13*^*C,* δ^*15*^*N and* δ^*34*^*S values)”*) accuracy was 85% (± 13.5%CV). Sulfur content in the lichen CRM is reported as an indicative value (2166 ± 292 mg kg^−1^; Quevauviller et al. 1996) a possible explanation for the less accurate IRMS analyses; batch-to-batch correction also were not undertaken for wt% S.

### Lichen stable-isotope-ratio ratios (δ^13^C, δ^15^N and δ^34^S values)

Ca. 7 mg lichen samples were weighed into tin capsules (6 × 4 mm), handled and closed with stainless steel tweezers and stored in 1.5 mL centrifuge tubes until analysis. Lichen stable-isotope-ratios were analysed by Continuous-Flow Isotope Ratio Mass Spectrometry (CF-IRMS) using an Elementar Pyrocube elemental analyser (EA) interfaced with a VisION isotope ratio mass spectrometer (IRMS) at the Scottish Universities Environmental Research Centre (SUERC), East Kilbride.

Stable-isotope ratios are expressed as ‘delta’ (δ) values, denoting measurement of isotope ratio relative to an accepted international reference frame (Eq. 1; Fry 2006; Sharp 2017), where:

1 $${\delta }^{y}X=\left(\frac{{R}_{sample}}{{R}_{standard}}-1\right)$$

Delta (δ) values are small numbers and are reported in per mille (‰), or parts per thousand (10^–3^; Coplen 2011), with y being the heavy isotope of element X (e.g., ^13^C) and R the ratio of abundance of the heavy to light isotope, for the sample (R_sample_) and the standard (R_standard_). In this study R is either ^13^C/^12^C (δ^13^C value), ^15^N/^14^N (δ^15^N value) or ^34^S/^32^S (δ^34^S value). A positive δ-value means that the ratio of the heavy to light isotope is higher in the sample compared to the standard, and the opposite for negative δ-values.

Recognised international reference standards were analysed alongside the lichen samples: USGS40 (glutamic acid; *N* = 8) for δ^13^C and δ^15^N, and silver sulphides (IAEA S2, *N* = 11, and S3, *N* = 11) for δ^34^S. Internal laboratory standards, MSAG2 (methanesulfonamide/gelatin; *N* = 121), M2 (methionine, gelatine, glycine and ^15^N-enriched alanine; *N* = 78) were used to monitor drift in δ^15^N, δ^13^C and δ^34^S values through time for each experimental batch (*N* = 3). IRMS accuracy varied between 98 and 102% for analysed reference standards (Table S2) and analytical precision for reference and internal standards was < 5%CV. Isotopic composition data are not available for lichen CRM BCR No. 482, but this reference material was measured throughout each analytical batch (*N* = 3) to check for potential analytical biases. Analytical precision (%CV) of repeated lichen CRM measurements (*N* = 43) was 2.4% (δ^13^C), 2.9% (δ^15^N) and 11% (δ^34^S), respectively. Batch-to-batch correction for lichen δ-values was not undertaken due to good repeatability (< 5%CV) for reference and internal standards across all measurements.

### Statistical and geospatial data analysis

Statistical analysis was completed using GraphPad Prism 7 and Minitab 17 statistical software (GraphPad Software Inc. 2018; Minitab 2019). Data visualisation was undertaken using Origin 2019 (OriginLab 2018) and R Studio with “ggplot2” package (RStudio Team 2021; Wickham 2016). Graphical visualisation was undertaken using QGIS 3.22.3 (QGIS Development Team 2022). Shapiro–Wilk test statistics were used to assess whether lichen CNS contents (wt%) and stable-isotope-ratios (δ-values) were normally distributed and inform subsequent statistical analysis, due to its strong statistical power regardless of data distribution and sample size (Razali and Wah 2011). Mann–Whitney tests (non-parametric; wt% C, δ^13^C and δ^34^S) or unpaired t-tests (wt% N, wt% S, δ^15^N) then were used to compare lichen species (*X. parietina* and *Physcia* spp.) and wt% CNS and stable-isotope ratio datasets.

**Fig. 2:**
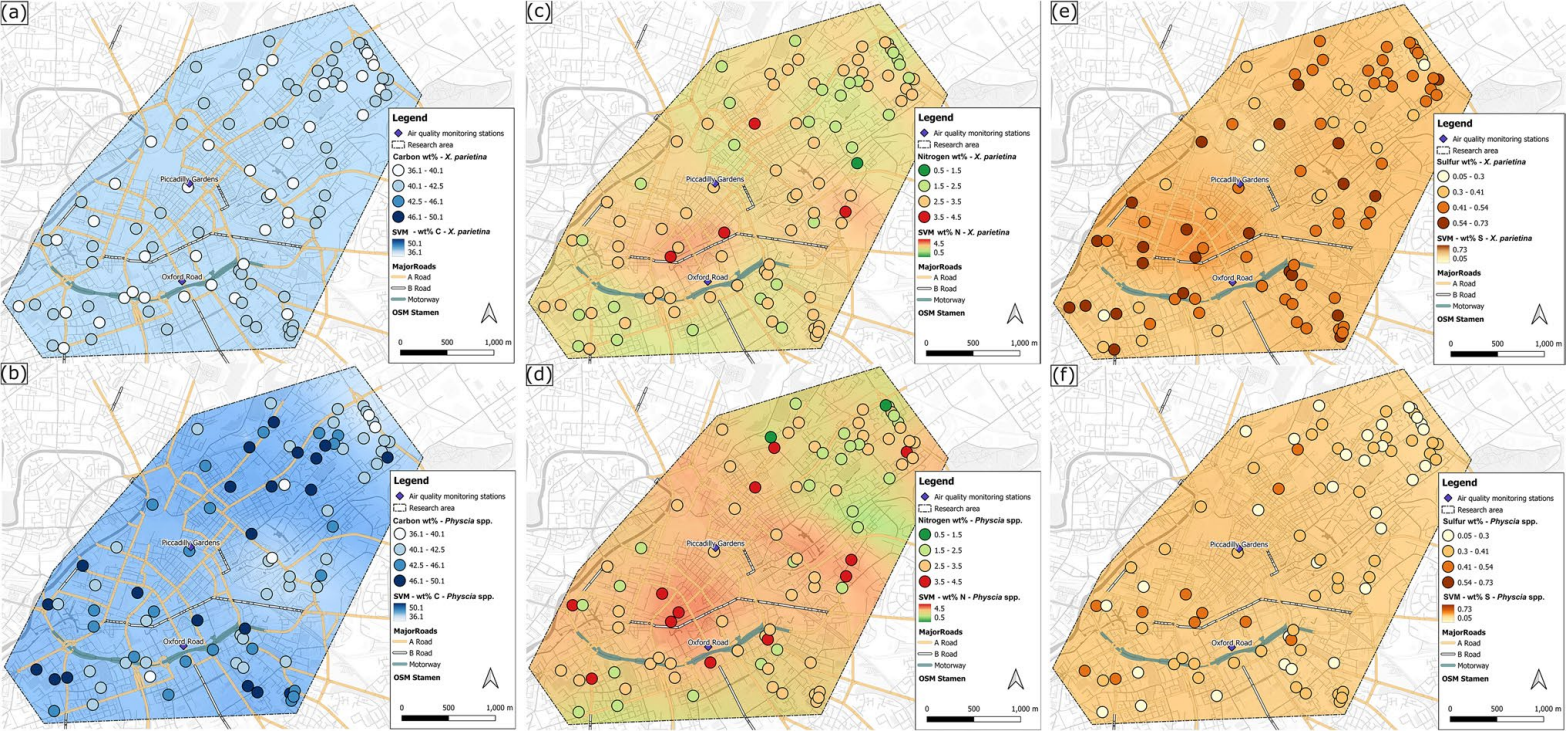
Lichen carbon [(**a**) and (**b**)], nitrogen [(**c**) and (**d**)] and sulfur contents [(**e**) and (**f**)] (wt%) in *X. parietina* and *Physcia* spp. colour-coded (low to high) across Manchester; displayed with automated air quality monitoring stations and major road network (A- and B-roads and motorway); and SVM interpolated values (same colouring and range) including Manchester’s urban factors

To investigate relationships between lichen-derived datasets and potential urban influencing factors, i.e. distance to major road, major road class, traffic counts, surrounding building heights and distance to green spaces, correlation statistics Pearson’s r (parametric) or Spearman ρ (non-parametric) were used, depending on the outcome of normal distribution of the dataset. Data sources for urban influencing factors and classification/grouping justifications are displayed in Table S3. These urban characteristics were used because they might impact on lichen pollutant loadings, by influencing dispersion, dilution and distribution of atmospheric pollutants. Additionally, urban influencing factors were mapped using an open-source machine learning interpolation method, Smart-Map plugin for QGIS (Pereira et al. 2022). The support vector machine (SVM) method can be used for regression and classification of small and large volumes of data (Karamizadeh et al. 2014; Pereira et al. 2022; Zhou et al. 2016). Urban factors (Table S3) were used as covariates for lichen chemical data in the SVM model, as well as X and Y-coordinates, (British National Grid), the value of the lichen data itself (i.e. CNS %wt and stable-isotope ratio signatures). Lichen values for CNS %wt and δ^13^C, δ^15^N and δ^34^S were and interpolated using Inverse Distance Weighting (IDW) withing the outlined research area (Fig. 2). Default IDW parameters were used, as described in Pereira et al. (2022), with a weighing value (p) as 1.00 for all covariables (i.e. urban factors), search radius equalling maximum distance between sampled points and number of neighbours set to 16. Moran’s Index (Moran’s I) was used for spatial correlation of regionalized variables, with univariate Moran’s I measuring the autocorrelation of the variable to be interpolated (i.e. lichen CNS wt% and stable isotope ratio signatures) and bivariate Moran’s I to measure spatial autocorrelation between covariates, i.e. Manchester’s urban factors. If the value is close or equal to zero, it means that the variable does not show spatial autocorrelation (Pereira et al. 2022). Moran’s I values closer to -1 or + 1 indicate greater spatial correlation of the variable (Pereira et al. 2022).

## Results and discussion

### Temporal variability of lichen CNS contents and impacts on assessment of spatial variability of lichen chemistry

Only carbon contents (wt% C) in repeat samples of *X. parietina* lichens (*N* = 18) were found to be significantly (*p* < 0.01) different, with elevated contents for samples collected in 2018, compared to 2016/17, whereas wt% N (*p* = 0.13) and wt% S (*p* = 0.53) were not significantly different (Fig. S4). The lack of any statistically significant differences for wt% N and wt% S suggest that lichen nitrogen and sulfur contents can be assumed to be reliable indicators of spatial variability of atmospheric nitrogen and sulfur compound concentrations, e.g., NO_2_ and SO_2_ did not vary significantly over this study’s period, and lichen wt% N and wt% S can reflect “localised” air quality. Albeit, elevated levels of NO_2_, including exceedances of the regulatory limit value (40 µg m^−3^), were recorded at both Manchester air quality monitoring stations, particularly during colder months (e.g. November 2016 to March 2017, Fig. S2; DEFRA 2018a, 2018b), such seasonal variation has not (significantly) impacted on lichen wt N% in this study. In contrast, consistent SO_2_ concentrations between 1.0–3.1 µg m^−3^ were recorded at Piccadilly Gardens between 2016 and 2018 (DEFRA 2018b). Indeed, SO_2_ concentrations in the UK have decreased by 96% since 1990 (NAEI 2018b)⁠ and no limit value exceedances, i.e. the WHO recommended daily mean value of 20 µg m^−3^ (WHO 2005, 2000) or EU limit value of 125 µg m^−3^ (daily mean; EU 2008)⁠, were recorded for Manchester (GMCA 2018)⁠. Therefore, lichen wt% S suggest localised impacts by atmospheric sulfur compounds, e.g. from domestic combustion, industrial manufacturing and energy production and transformation (DEFRA 2022) in Manchester.

On the contrary, no data for carbon compounds (e.g., CO and CO_2_) is available for Manchester’s automated air quality stations (for this study’s period). However, carbon (i.e., elemental and organic) is a major component of particulate matter (i.e. PM_10_) in urban centres across the UK, derived from incomplete combustion of organic material and vehicle tyre wear (Harrison et al. 2004; Jones and Harrison 2005; Rogge et al. 1993). In this study, lichen samples were not washed prior to analysis and increased wt% C could be due to increased number of entrapped particulates on the lichen surface, when re-sampling was undertaken in 2018. However, differences in lichen wt% C also could be related to microclimatic conditions, bark pH and lichen species-specific compounds, such as genus-specific sugar alcohols that are used by the mycobiont (Beck and Mayr 2012; Gaio-Oliveira et al. 2005a; Hauck 2010; Máguas et al. 2013). Hence, increased wt% C in 2018 could be linked to fungal metabolism, induced by higher availability of nutrients, in particular nitrogen and sulfur (Gaio-Oliveira et al. 2005a). Investigating lichen chlorophyll contents, amino acids and secondary lichen products (of fungal origin; Ranković and Kosanić, 2019)⁠ could provide further insights into the causes of increased carbon contents and potentially explain better the observed temporal variability (Gaio-Oliveira et al. 2005a; Hauck 2010).

The lichen wt% N and wt% S content results suggest a potential ‘equilibrium’ (i.e. balance) state of *X. parietina* with its close surrounding environment (Paoli et al. 2018); i.e. reflecting the local atmospheric conditions at the sampled locations. Nonetheless, seasonal variability of atmospheric pollutants has been reported for urban environments in Europe and the UK (Hernández-Paniagua et al. 2015; Masiol et al. 2017; Vardoulakis et al. 2011), which is also recorded by automated air quality measurements in Manchester (Fig. S2). The results of this study suggest that lichen carbon content at individual locations can vary over multiple-year timescales, in response to temporally variable air quality, and hence cannot be linked to atmospheric carbon composition, and hence air quality, exclusively. In contrast, wt% N and wt% S do not vary over such timescales, due to likely non-significant changes in pollutant concentrations (at least in Manchester) and thus can be considered robust measurements to assess spatial variability in urban air quality.

### Spatial variability of carbon, nitrogen and sulfur contents in urban lichens (*X. parietina* and *Physcia* spp.) to evaluate potential pollution sources

Lichen CNS contents were found spatially variable across Manchester (Fig. 2), with higher variability in wt % N (Fig. 2 c and d) and S wt% (Fig. 2 e and f), compared to wt% C (Fig. 2 a and b), in both lichen species. Individual lichen data for sampling locations are displayed in the supplementary material (Table S4 and Fig. S5).

Besides spatial variability, species-specific differences in recorded CNS wt% (Fig. S6) were recorded, suggesting potential different suitability of *X. parietina* (*N* = 94) and *Physcia* spp. (*N* = 87) lichens for (urban) biomonitoring approaches. Such species-specific differences between *X. parietina* and *Physcia* spp. (Fig. S6) could be related to different assimilation abilities of nutrients, e.g. N and S compounds, and subsequent differences in biomass increases and net CO_2_ gain (Dahlman et al. 2003; Johansson et al. 2010). However, 31% of CO_2_ in Manchester is emitted by road traffic (TfGM and GMCA 2016), which could be related to increased wt% C in *Physcia* spp. at some locations (Fig. 2 b). In contrast, temporal variability was recorded in *X. parietina* (“Temporal variability of lichen CNS contents and impacts on assessment of spatial variability of lichen chemistry”), suggesting a potential bias on lichen wt% C variability. Consequently, lichen wt% C data do not provide a beneficial tool for assessment of spatial variability of airborne carbon compounds in an urban environment.

Urban environments are complex systems, comprising rough surfaces that influences the aerodynamic properties of the atmosphere, which are critical for air pollutant deposition and dispersion (Cariolet et al. 2018; Grimmond and Oke 1999). Lichen CNS contents (and stable-isotope ratio signatures; “Spatial variability of lichen stable-isotope-ratio signatures and assessment of pollution source apportionment in the Manchester urban environment”) were considered in relation to different potential urban influencing factors that may impact on dispersion and distribution of pollutants (i.e., major road distances and surrounding building heights), and thus allowing investigation of air quality at the sampling location in finer detail. Correlation statistics for lichen data and potential urban influences (as described in Table S3) are displayed in Table 1. Table 2 illustrates the spatial autocorrelation (Moran’s I) between the lichen dataset and potential urban influencing factors across Manchester.

**Table 1:** Correlation statistics (Pearson's r and Spearman ρ) used to investigate relationships between urban influencing factors and lichen-derived CNS contents (wt%) and stable-isotope-ratio signatures (δ^13^C, δ^15^N and δ^34^S). Underlined values represent Spearman ρ, all other values are presented as Pearson’s r, significant values are presented in bold (significance level is indicated by asterisk; *significant at the level *p* < 0.05 and **significant at the level *p* < 0.01); MR = distance to major road, RdCl = road class, TC = traffic counts, BH = surrounding building height, GS = distance to greenspace

	MR	RdCl	TC	BH	GS
*X. parietina*					
wt% C	0.11	0.01	-0.12	-0.17	-0.07
wt% N	-0.21*	0.19	0.07	0.30**	0.17
wt% S	-0.13	0.12	0.11	0.30**	0.12
δ^13^C	0.22*	-0.26*	-0.07	-0.20	-0.21*
δ^15^N	-0.28**	-0.34**	0.22*	0.25*	0.20
δ^34^S	0.15	-0.01	-0.14	-0.21*	-0.08
*Physcia* spp.					
C wt%	-0.26	0.00	-0.11	-0.02	0.00
N wt%	-0.14	0.07	0.24*	0.16	0.16
S wt%	-0.19	0.07	0.25*	0.16	0.16
δ^13^C	0.20	-0.11	-0.13	-0.08	-0.15
δ^15^N	-0.26*	0.21	-0.26*	0.09	0.27*
δ^34^S	0.22*	0.04	-0.29**	-0.21	-0.10

**Table 2:** Moran’s I (ranging from -1 ≤ to ≤  + 1) to evaluate spatial autocorrelation (i.e. how similar objects are to others surrounding it) between urban influencing factors and lichen-derived CNS contents (wt%) and stable-isotope-ratio signatures (δ^13^C, δ^15^N and δ^34^S); significant values are presented in bold (significance level is indicated by asterisk: *significant at the level *p* < 0.05); MR = distance to major road, RdCl = road class, TC = traffic counts, BH = surrounding building height, GS = distance to greenspace

	MR	RdCl	TC	BH	GS
*X. parietina*					
wt% C	0.07	-0.07	-0.09*	-0.08*	-0.10*
wt% N	-0.12*	0.08	0.09*	0.22*	0.15*
wt% S	-0.07	0.05	0.10*	0.22*	0.10*
δ^13^C	0.09	-0.06	-0.13*	-0.17*	-0.16*
δ^15^N	-0.11*	0.09*	0.19*	0.18*	0.18*
δ^34^S	0.09	-0.05	-0.11*	-0.20*	-0.68
*Physcia* spp.					
C wt%	0.06	-0.35	-0.02	-0.02	-0.02
N wt%	-0.10*	0.05	0.17*	0.14*	0.16*
S wt%	-0.10*	0.05	0.17*	0.16*	0.16*
δ^13^C	0.04	0.04	-0.10*	-0.16*	-0.13*
δ^15^N	-0.11*	0.07	0.20*	0.06	0.22*
δ^34^S	0.13*	-0.08	-0.17*	-0.23*	-0.08

Recorded *X. parietina* and *Physcia* spp. carbon contents from the Manchester urban environment are comparable to results presented for Naples, Italy (Vingiani et al. 2004)⁠ and interestingly, also a remote Antarctic location (King George Island; Lee et al. 2009). In this study, elevated wt% C were recorded for *Physcia* spp. within the city centre area and along the major road network (Fig. 2 b), whereas such a pattern was not visible for *X. parietina* (Fig. 2 a). Carbon wt% in *X. parietina* and *Physcia* spp. showed no significant correlation (Spearman ρ, *p* > 0.05) with Manchester’s urban factors (Table 1), whereas significant spatial correlation (*p* < 0.05) in *X. parietina* wt% C was recorded with urban factors (Table 2), indicating spatially dispersed data with potential site-specific influences, e.g. from traffic counts and surrounding building heights and aforementioned sampling location characteristics (i.e. tree bark).

The variability of nitrogen contents in *X. parietina* sampled from across Manchester was primarily influenced by the sampling locations distance to a major road (A-, B- roads and motorway; *p* < 0.05; Table 1, Fig. S7) and surrounding building height (*p* < 0.01; Table 1, Fig. S7). In contrast, no significant relationships for these variables were found for *Physcia* spp., whereas traffic counts were positively correlated (*p* < 0.05) with wt% N. Consequently, elevated traffic at sampling locations suggests impacts by atmospheric nitrogen compounds, e.g. from vehicular sources (NO_x_ and NH_x_). For instance, within Manchester 80% of NO_x_ emissions are related to vehicular emissions, which have previously been reported to impact on lichen wt% N (Bermejo-Orduna et al. 2014; Gombert et al. 2003; Regan 2018). Moreover, Niepsch et al. (2021a) reported the results of a detailed diffusion-tube monitoring study and elevated NO_2_ concentrations within Manchester’s city centre and along its major road network. Measured diffusion tube NO_2_ concentrations were significantly positive correlated with wt% N (Pearson’s r = 0.34, *p* < 0.05), with higher wt% N where atmospheric NO_2_ concentrations where elevated.

Major roads, e.g. motorways and A roads, are often the main arteries within urban environments and usually have high traffic flows (DfT 2017) and it is well known that pollutants, such as vehicular NO_2_, decrease rapidly with distance from a major road/source and that densely built-up urban centres with larger and wider buildings impact on pollutant distribution and dispersion (Cyrys et al. 2012; Kubota et al. 2008; Kurppa et al. 2018). In contrast, vegetated green spaces and sparsely built-up areas can have a positive impact on urban air quality (Britter and Hanna 2003; Janhäll 2015; Salmond et al. 2013) that could explain some of the recorded differences in lichen wt% N across Manchester.

Moreover, lichen wt% N showed significant (*p* < 0.05, Table 2) spatial correlation with urban factors, i.e. distance to highly-trafficked roads (MR and TC), distance to greenspace (GS) and surrounding building heights (BH). Hence, displaying clusters (e.g. the city centre area; Fig. 2) of elevated N wt% being likely influenced by local sources (e.g. vehicular emissions) and poor dispersion of air pollutants within the densely built-up city centre area. Comparable results were reported for lichen biomonitoring studies (incorporating *X. parietina* and/or *Physcia* spp.) undertaken in urban, agricultural and industrial regions of Europe; e.g., in the Ruhr-Area and South Germany (Beck and Mayr 2012; Boltersdorf and Werner 2013; Franzen-Reuter 2004), Grenoble, France (Gombert et al. 2003) and the Alentejo Litoral region, Portugal (Pinho et al. 2017).

Nonetheless, urban airborne nitrogen compounds comprise different nitrogen compounds (e.g., nitrate, ammonia and ammonium – NO_x_ and NH_x_) that presumably can affect nitrogen deposition (wet and dry; Van Herk 2003) and consequently lichen wt% N. Albeit, lichen total wt% N can reflect airborne nitrogen loads, such an approach cannot distinguish between different nitrogen compounds, i.e. nitrate (NO_3_^−^) and ammonium (NH_4_^+^). Accordingly, different nitrate (NO_3_^−^) and ammonium (NH_4_^+^) concentrations have been reported in *X. parietina* samples across Manchester (Niepsch et al. 2021b), suggesting site-specific and diverse sources; e.g., from vehicular, domestic and industrial emissions that potentially impacted on lichen wt% N recorded by this study (further discussed in “*Lichen nitrogen stable-isotope ratios (δ*^*15*^*N)*”**;** Fig. 8). Nonetheless, lichen nitrogen contents can provide beneficial information on atmospheric nitrogen compounds across an urban environment, and thus identify potential hotspots of poor air quality and associated deleterious negative health impacts, certainly not distinguishing between potential sources.

For both lichen species, wt% S was elevated within the city centre, whereas lower contents were recorded in more residential areas (northeast and southwest of the research area; Fig. 2 d and e). However, wt% S in both lichen species was spatially correlated with urban factors (TC, BH and GS; Table 2) illustrating the clustering of (similar) data. For example, elevated wt% S within the city centre area (in *X. parietina*, Fig. 2 e) could be related to sulphate-containing particulates entrapped on the lichen surface, which derive from domestic sources (e.g. wood and coal fires and stoves) and vehicular emissions, e.g. diesel-powered vehicles (AQEG 2012; Clean Air Greater Manchester 2021; Zereini and Wiseman 2011). Moreover, SO_2_ is an important precursor for PM_2.5_ formation (AQEG 2012) and elevated wt% S in lichens could indicate areas with elevated PM_2.5_ concentrations, and thus poor air quality and potential human health impacts (e.g. cardiovascular and respiratory diseases; Regan 2018). Investigating the sulfur content of potential sulfur sources (e.g. petroleum coke used in the UK, NAEI 2018b) and particulate matter (e.g. PM_10/2.5_) would further refine understanding of the potential influences on elevated lichen wt% S.

Recorded sulfur contents in lichen species sampled across Manchester were higher compared to previous urban lichen wt% S studies, that used transplanted *Usnea amblyoclada* and *Ramalina ecklonii* from Cordoba City, Argentina (Carreras and Pignata 2002; González et al. 1996) and *Alectoria sarmentosa* from Newfoundland, Canada (Wadleigh 2003; Wadleigh and Blake 1999; Wiseman and Wadleigh 2002). Such differences are most likely linked to the different study designs (i.e. active and passive biomonitoring [this study]), the utilised lichen species (e.g. fruticose and foliose [this study]) and subsequent species-specific sensitivity towards atmospheric pollutants, with fruticose lichens generally being more sensitive to atmospheric pollution, compared to foliose (and crustose) lichens (Shukla et al. 2014)⁠. Moreover, sulfur is an essential lichen nutrient (Wiseman and Wadleigh 2002), such that differences observed between the *X. parietina* and *Physcia* spp. utilised in this study could be related to species-specific sensitivity to high concentrations of sulfur compounds. For instance, *X. parietina* is reportedly less affected by elevated ambient pollutant concentrations (Dobson 2011; Kirschbaum and Wirth 2010). Further, the geographic region and specific elevated regional pollution patterns could explain higher wt% S recorded for Manchester. For instance, SO_2_ pollution in Manchester is primarily linked to anthropogenic sources, i.e. industrial processes and combustion from energy industry (Manchester City Council 2018), which is comparable to results presented by lichen transplant studies, i.e. local industrial sources (Carreras and Pignata 2002; Wadleigh 2003; Wadleigh and Blake 1999).

Supplementary datasets, e.g. traffic counts on minor roads, pollutant dispersion and distribution from large point sources and interception by urban vegetation (e.g. uptake of gaseous, aerosol and particulate pollutants; Freer-Smith et al. 1997; Gaston et al. 2010) are scarce. Data classification used in this study were based on available (limited) datasets and grouping justification was based on urban air quality and pollution studies reporting the decline of pollutants (e.g. NO_2_ within 200 m of highly-trafficked roads: Bermejo-Orduna et al. 2014; Gombert et al. 2003; Laffray et al. 2010) and impact of urban density on dispersion and distribution of pollutants (Table S3). Nonetheless, they were considered useful to investigate the distribution (e.g. from major roads and road traffic), dispersion (e.g. building heights) and spatial variability of pollutants within the urban environment of Manchester and used for geospatial interpolation (Fig. 2). However, due to the complexity of urban environments and additional meteorological influences, such as temperature and rainfall, atmospheric processes and other atmospheric pollutants as well as sampling location-specific influences (e.g. bark pH), lichen wt% CNS also could be related to those additional factors and not just to vehicle exhaust emissions. These factors should be included in future studies to assess spatial variability of air quality in more depth and to also potentially to help pinpoint pollution sources. Overall, lichen wt% N and wt% S have been shown to have been impacted by their site-specific surroundings using an IDW-based machine learning algorithm (SVM, Pereira et al. 2022; Table 2 and Fig. 2).

In conclusion, elevated lichen wt% N and wt% S can be used to document spatial variability (i) and to identify locations with deteriorated air quality (for N and S compounds) and thus identify areas of human health concern that require ameliorative actions. Although, these data alone cannot be used to identify potential airborne pollution sources, whereas lichen stable-isotope-ratio signatures (δ^13^C, δ^15^N and δ^34^S values) have potential utility for source apportionment. It is further suggested to utilise the same lichen species across a sampled area, specifically when the aim is to compare (different) environments, because of the species-specific differences in lichen CNS contents identified by this study (that used foliose lichen *X. parietina* and *Physcia* spp.; Fig. S6 a-c). For instance, *X. parietina* is a good choice for biomonitoring or urban air quality, because of its wide distribution and ability to thrive in nitrogen-rich environments (Olsen et al. 2010; Pinho et al. 2008).

### Spatial variability of lichen stable-isotope-ratio signatures and assessment of pollution source apportionment in the Manchester urban environment

#### Lichen carbon stable-isotope ratios (δ^13^C)

**Fig. 3:**
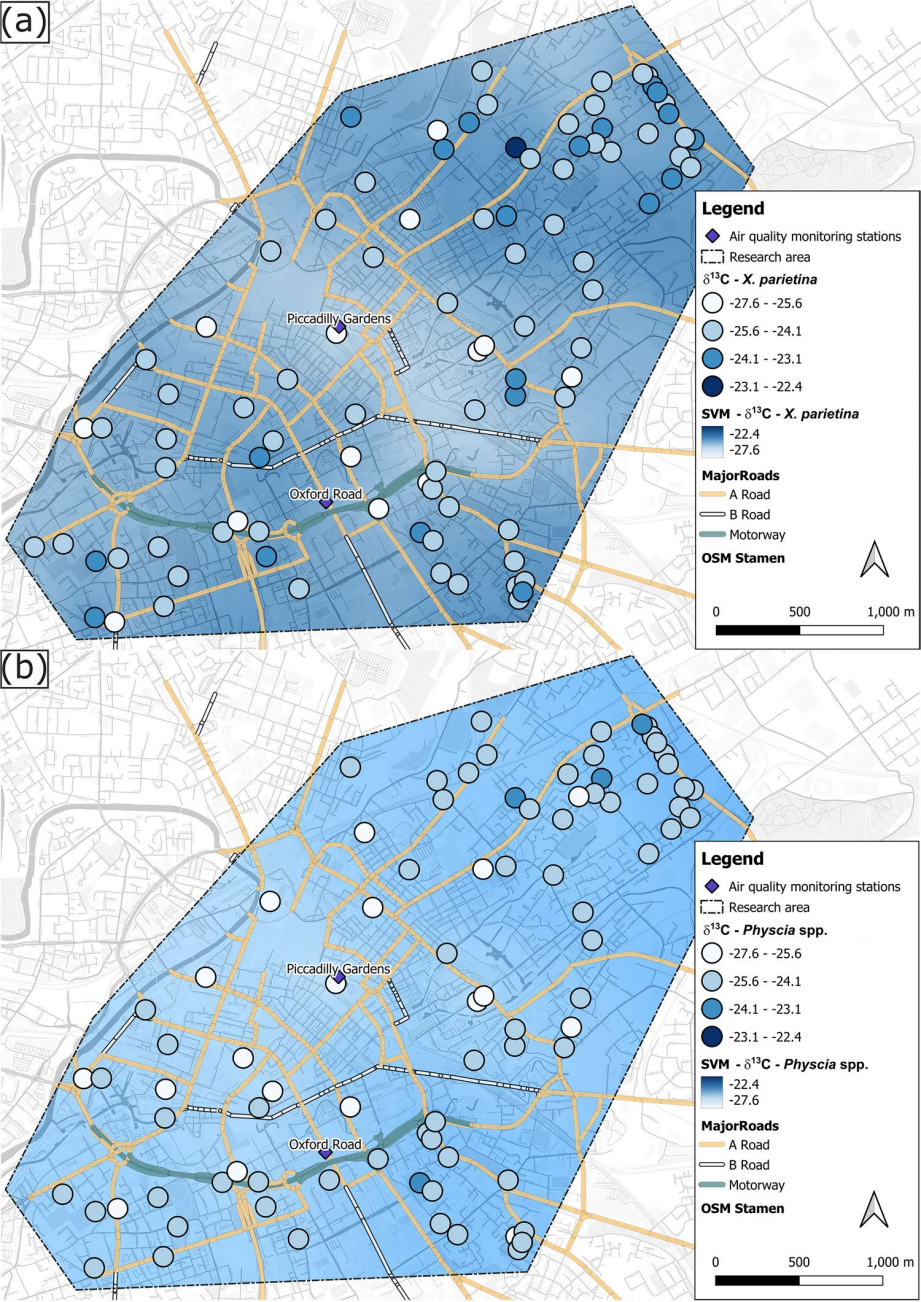
δ^13^C values (‰) of (**a**) *X. parietina* and (**b**) *Physcia* spp. colour-coded by ^13^C-enrichment (more positive values) across Manchester; displayed with automated monitoring stations and major road network (A, and B-road and motorway); displayed with SVM interpolated values (same colouring and range) including Manchester’s urban factors

Lichen stable carbon-isotope ratio signatures were found to be variable across Manchester, with more variable δ^13^C-values in *X. parietina* (-26.7 to -22.4‰; Fig. 3 a) compared to *Physcia* spp. (-27.6 to -23.6‰; Fig. 3 b). A significant (*p* < 0.05) negative relationship between wt% C and δ^13^C for *Physcia* spp. (Fig. 1; Table S6) was recorded, whereas no significant relationship was found for *X. parietina*. Spatial clustering of lichen δ^13^C-values and urban factors (TC, BH and GS, *p* < 0.05; Fig. 3), was recorded, with enriched ^13^C contents in more residential surroundings and along the major road network (Fig. 3), suggesting localised influences. However, green-algal lichens, including *X. parietina* and *Physcia* spp., follow the C_3_ photosynthetic pathway and generally have δ^13^C values of -28‰ following fractionation of carbon isotopes during uptake of atmospheric CO_2_ (Batts et al. 2004). Cities are centres of concentrated CO_2_ emissions (Mitchell et al. 2018) and seasonal variations of CO_2_ and δ^13^C-CO_2_ have been reported in European urban environments; e.g. London (UK), Krakow (Poland), Wrocław (Poland) and Bern (Switzerland) (Górka and Lewicka-Szczebak 2013; Hernández-Paniagua et al. 2015; Pazdur et al. 2013; Sturm et al. 2006; Zimnoch et al. 2004). In this study, temporal variability was recorded for lichen wt% C whereby spatial variability of lichen δ^13^C values also might have been impacted by temporal variations in urban CO_2_ concentrations and its δ^13^C signature.

**Fig. 4:**
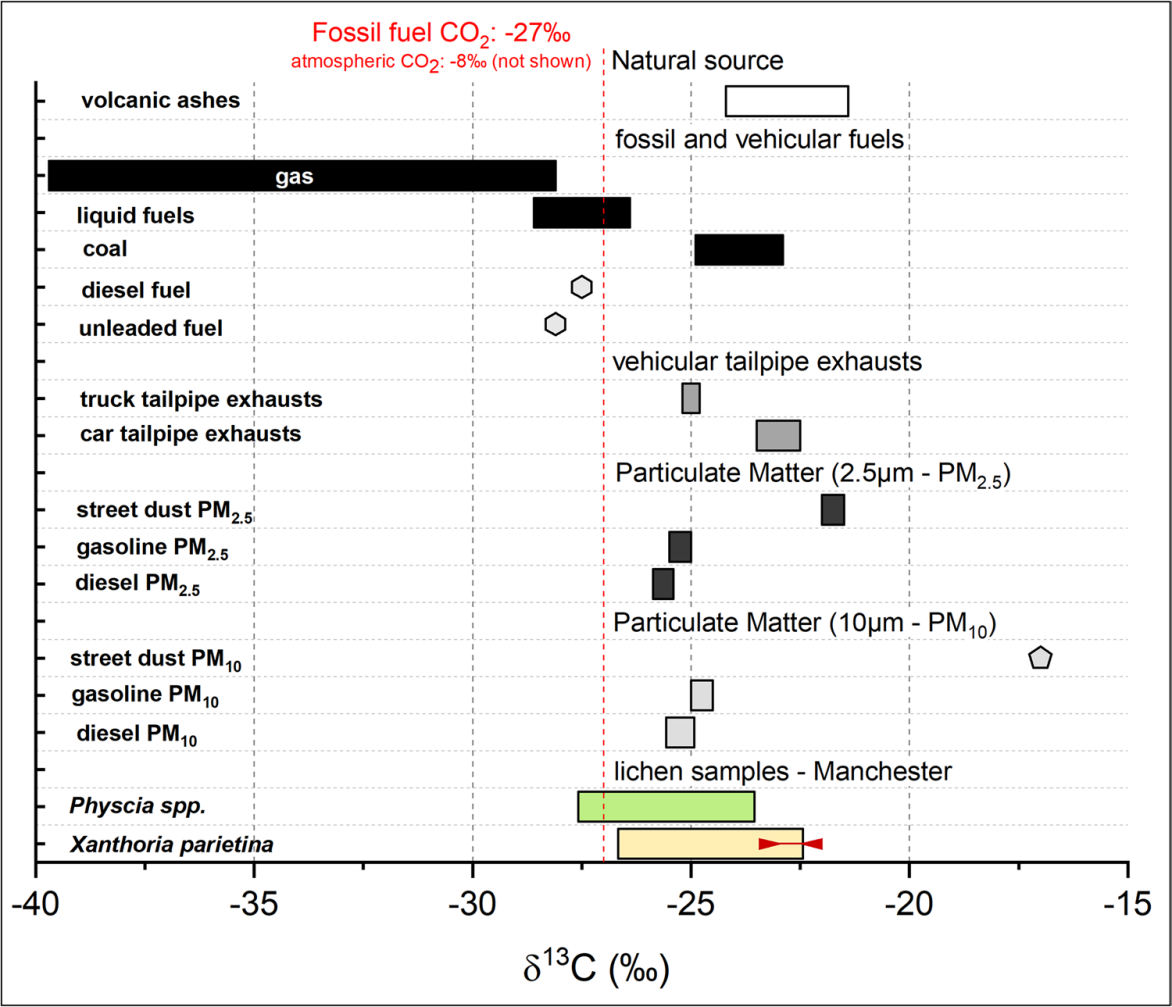
δ^13^C values of potential atmospheric carbon pollutants and pollution sources reported in the literature (Górka et al. 2020; López-Veneroni 2009; Widory 2007, 2006) to undertake lichen δ^13^C value source apportionment; displayed with δ-values of lichens (*X. parietina* and *Physcia* spp.) sampled across Manchester (rural *X. parietina* are displayed as red solid line on bar for comparison)

Figure 4 shows the stable-isotope compositions for different potential natural and anthropogenic carbon airborne pollution sources, against which comparisons can be made when attempting source apportionment.

Manchester lichen δ^13^C values are comparable to prior air quality studies in the urban area of Sydney (Australia) and Mexico City (Mexico), that reported street dust PM_10_, diesel soot and gasoline vehicle exhausts impacting on lichen δ^13^C values (Batts et al. 2004; López-Veneroni 2009). For instance, Górka et al., (2020) reported PM_10-_δ^13^C in ranges of -24.3 to -26.8‰ at an urban site in Poland. Lichen lichen-δ^13^C span δ^13^C-PM ranges (Fig. 4), suggesting a potential impact of deposited particulates on the lichen surface, because lichens were not washed prior to analysis. However, because PM-δ^13^C and other anthropogenic sources (Fig. 4) also span different δ^13^C values (Fig. 4), a clear distinction of solely anthropogenic sources is not possible using lichen stable carbon-isotope signatures.

**Fig. 5:**
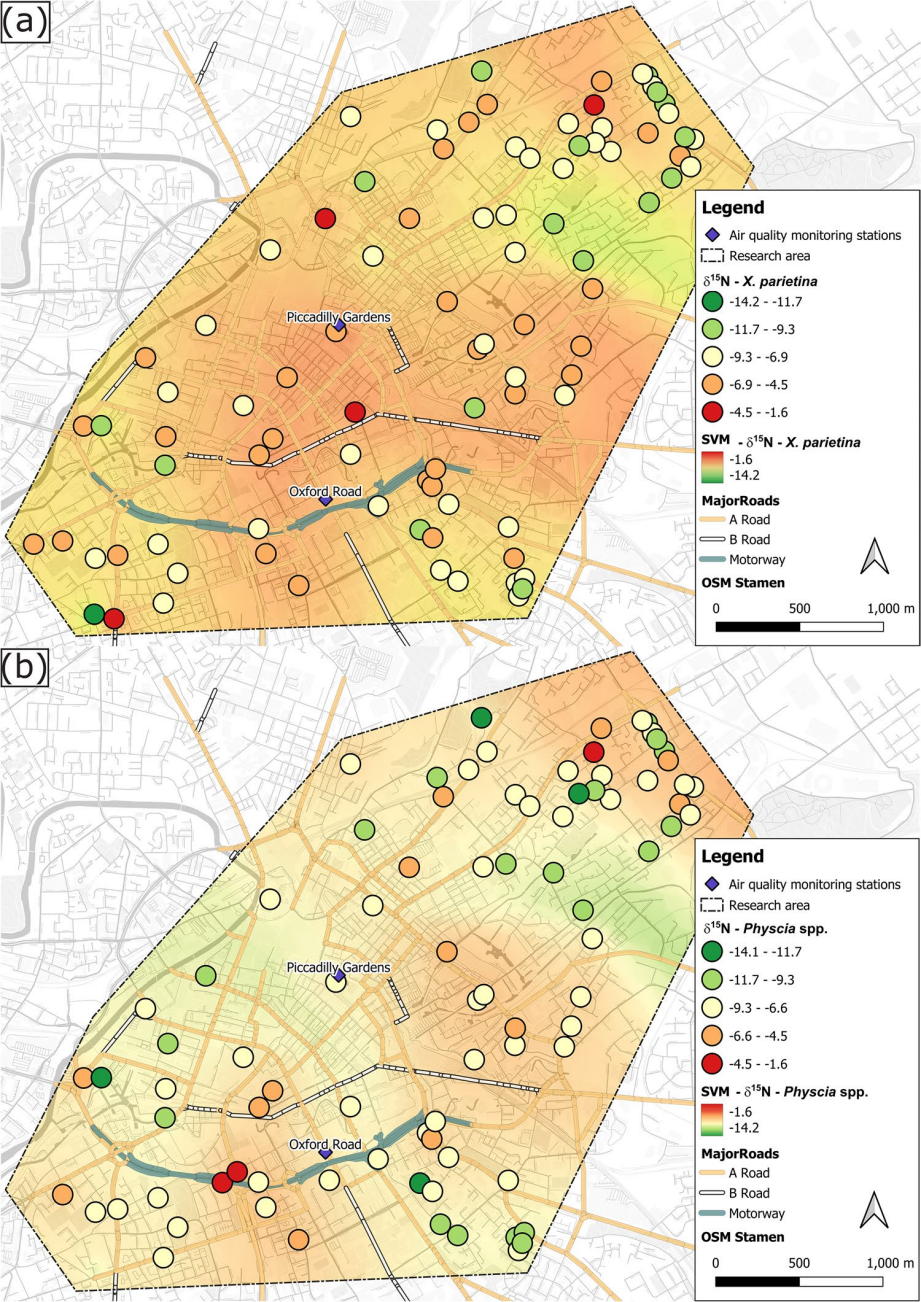
δ^15^N values (‰) of (**a**) *X. parietina* and (**b**) *Physcia* spp. colour-coded by ^15^N-enrichment (more positive values) across the City of Manchester; displayed with automated monitoring stations and major road network (A- and B-road and motorway); displayed with SVM interpolated values (same colouring and range) including Manchester’s urban factors

Alternatively, minor variation recorded in δ^13^C values (and carbon contents; “*Spatial variability of carbon, nitrogen and sulfur contents in urban lichens (X. parietina and Physcia spp.) to evaluate potential pollution sources*”) for both Manchester lichens are potentially linked to different types of sources emitting atmospheric carbon, related δ^13^C ranges and meteorological parameters (e.g. water and light availability), physiological and/or biochemical processes (e.g. assimilation, metabolism and biosynthesis; Batts et al. 2004; Beck and Mayr 2012; Biazrov 2012; Ciężka et al. 2022; Galimov 2000; Hoefs 2009; Lee et al. 2009), as well as other atmospheric pollutants. For instance, Batts et al. (2004) reported decreasing δ^13^C with increasing pollution levels, i.e. when NO_x_ (and SO_2_) increases. In this study, increased atmospheric nitrogen compounds, as suggested by elevated lichen wt% N (“*Spatial variability of carbon, nitrogen and sulfur contents in urban lichens (X. parietina and Physcia spp.) to evaluate potential pollution sources*”), potentially could have had a similar impact. To evaluate such impacts, lichen wt% C and wt% N, as well as stable carbon and nitrogen isotope ratios were correlated with one another (Table S6). No statistically significant differences between wt% C and wt% N were recorded for *X. parietina,* whereas δ^15^N and δ^13^C were significantly negatively (*p* < 0.05) correlated (Table S6), i.e. δ^13^C (Fig. 3) was more negative at sampling locations in proximity to major roads, which also showed an enrichment in ^15^N (Fig. 5). Moreover, Niepsch et al. (2021a) recorded elevated NO_2_ concentrations (by passive sampling devices) at locations with high wt% N. Hence, a potential impact on lichen δ^13^C by elevated nitrogen (and potentially sulfur) concentrations are suggested that require further investigation. However, statistically significant differences between lichen isotope-ratio signatures also be linked to differences in ambient air chemistry in the investigated environments. Investigating isotopic signatures of carbon compounds (among others, e.g. NO_x_, NH_x_ and SO_x_) and particulate matter (PM_2.5/10_) will aid to fully understand influences on lichen carbon-isotope-ratio signatures.

In summary, Manchester lichen δ^13^C values, as well as wt% C, are not considered reliable indicators of sources of different airborne pollutant carbon compounds in the urban environment (ii), an outcome in accordance with Bermejo-Orduna et al. (2014) who studied the lichen *Letharia vulpina* along a highly-trafficked motorway in Nevada (USA).

#### Lichen nitrogen stable-isotope ratios (δ^15^N)

Spatial variability of δ^15^N values was recorded for both lichen species (Fig. 5), ranging between -13.6 and -1.6‰ (*X. parietina*) and -14.2 and -2.0‰ (*Physcia* spp.) across Manchester. For both lichen species, an increase in wt% N was significantly (*p* < 0.01) accompanied by an enrichment in ^15^N (Fig. S5; Table S6). Comparably, statistically significant correlation of lichen-δ^15^N with urban influencing factors, as well as spatial correlation (Table 2, Fig. 5) with these factors suggest localised sources of pollution in Manchester.

**Fig. 6:**
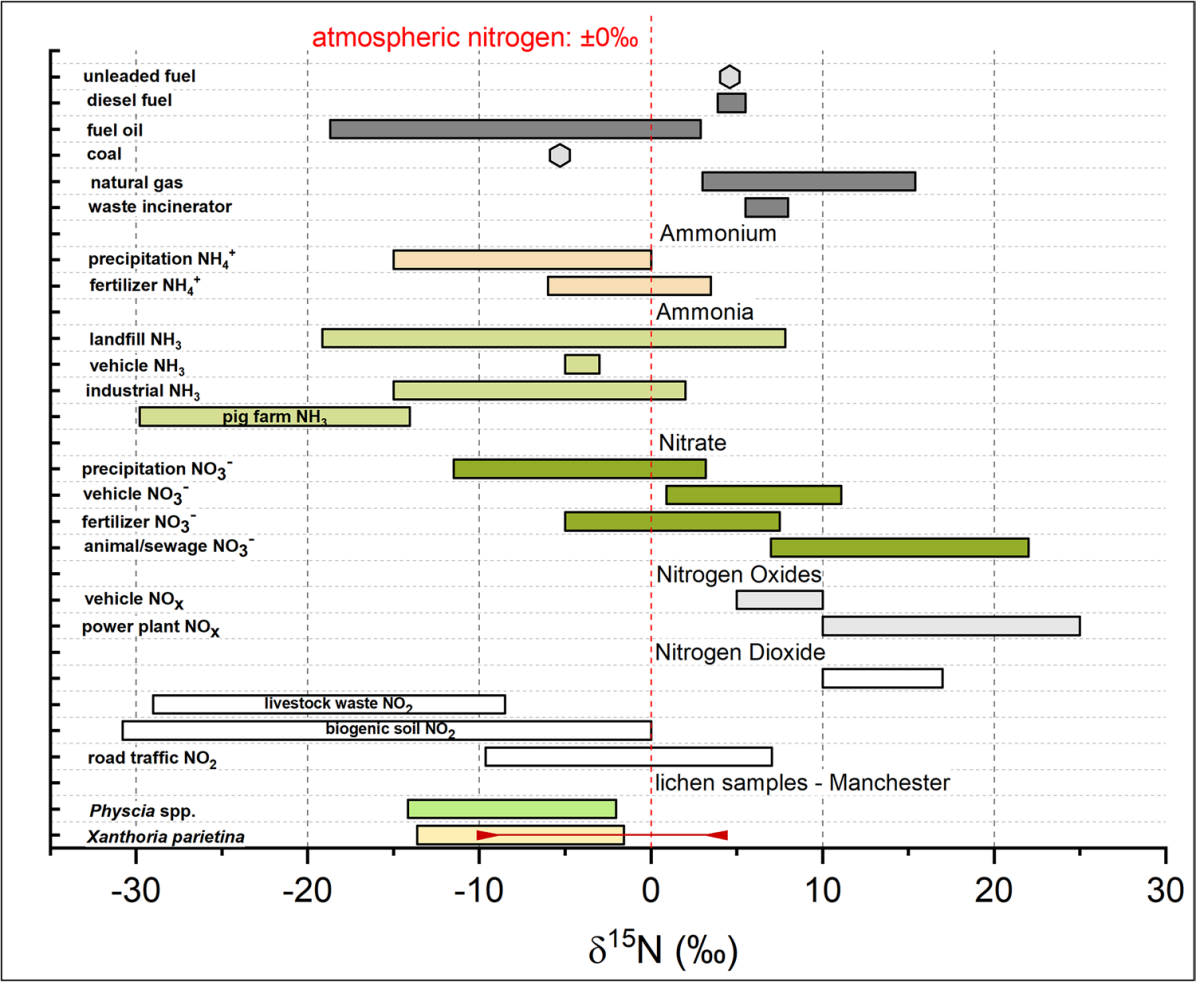
δ^15^N values of potential atmospheric pollutants and pollution sources reported in the literature (Berner and Felix 2020; Elliott et al. 2007; Felix et al. 2013, 2012; Felix and Elliott 2014; Heaton 1986; Heaton et al. 1997; Liu et al. 2016) to undertake lichen δ^15^N value source apportionment; displayed with δ-values of lichens (*X. parietina* and *Physcia* spp.) sampled across Manchester (rural *X. parietina* are displayed as red solid line on bar for comparison)

Epiphytic lichens can exhibit a wide range of δ^15^N values, due to assimilation of nitrogen compounds from different sources (e.g., NO_x_ and NH_x_; Beck and Mayr 2012). For instance, lichen δ^15^N values have been reported to be more positive where airborne NO_x_ concentration is high, such as in urban areas, and more negative in rural areas, the latter primarily due to a greater contribution of reduced nitrogen (NH_x_) from agricultural sources (Boltersdorf et al. 2014; Pearson et al. 2000; Sutton et al. 2004). Figure 6 summarises δ^15^N ranges of anthropogenically-emitted nitrogen compounds, with this study’s *X. parietina* and *Physcia* spp. lichen δ^15^N values spanning a variety of potential sources, including different atmospheric NH_x_ and NO_x_ compounds.

Albeit covering a variety of potential sources (Fig. 6), Manchester *X. parietina* and *Physcia* spp. δ^15^N values (as well as wt% N for both lichen species: Fig. 2) were more positive along highly-trafficked major road network (Table 1, Fig. 5) and suggesting a primary NO_x_ influence on nitrogen isotope signatures. Indeed, lichen enrichment in ^15^N was recorded at sampling locations in proximity to the highly-trafficked Mancunian Way with approximately 30,000 vehicles passing daily (DfT 2017). Comparably, δ^15^N was positively correlated with passively-derived NO_2_ concentrations by Niepsch et al. (2021a) in *X. parietina* (Pearson’s r = 0.54, *p* < 0.01) and *Physcia* spp. (Pearson’s r = 0.37, *p* < 0.01; Table S6), consequently suggesting a major NO_x_-related influence in Manchester. Such findings have been reported by Bermejo-Orduna et al. (2014) who analysed the lichen *Letharia vulpina* δ^15^N along a major highway (I 80) in California (USA) and by Laffray et al. (2010) and Pearson et al. (2000) who used mosses as biomonitors along main routes (RN6 and A43) in the Maurienne Valley (France) and the urban environment of London (UK), respectively. On the contrary, Pinho et al. (2017) reported more negative δ^15^N values (-15.59‰ to -11.24‰) in *Parmotrema hypoleucinum*, sampled in the urban area of Sines (Portugal), linked to varying sources of NO_x_ and NH_x_. Indeed, differences in recorded δ^15^N-values could be related to the species used in these biomonitoring studies, it is generally accepted that δ^15^N of epiphytic lichens reflect existing nitrogen compounds from the atmosphere, allowing analysis of predominant nitrogen sources (Boltersdorf and Werner 2014; Fogel et al. 2008; Tozer et al. 2005).

**Fig. 7:**
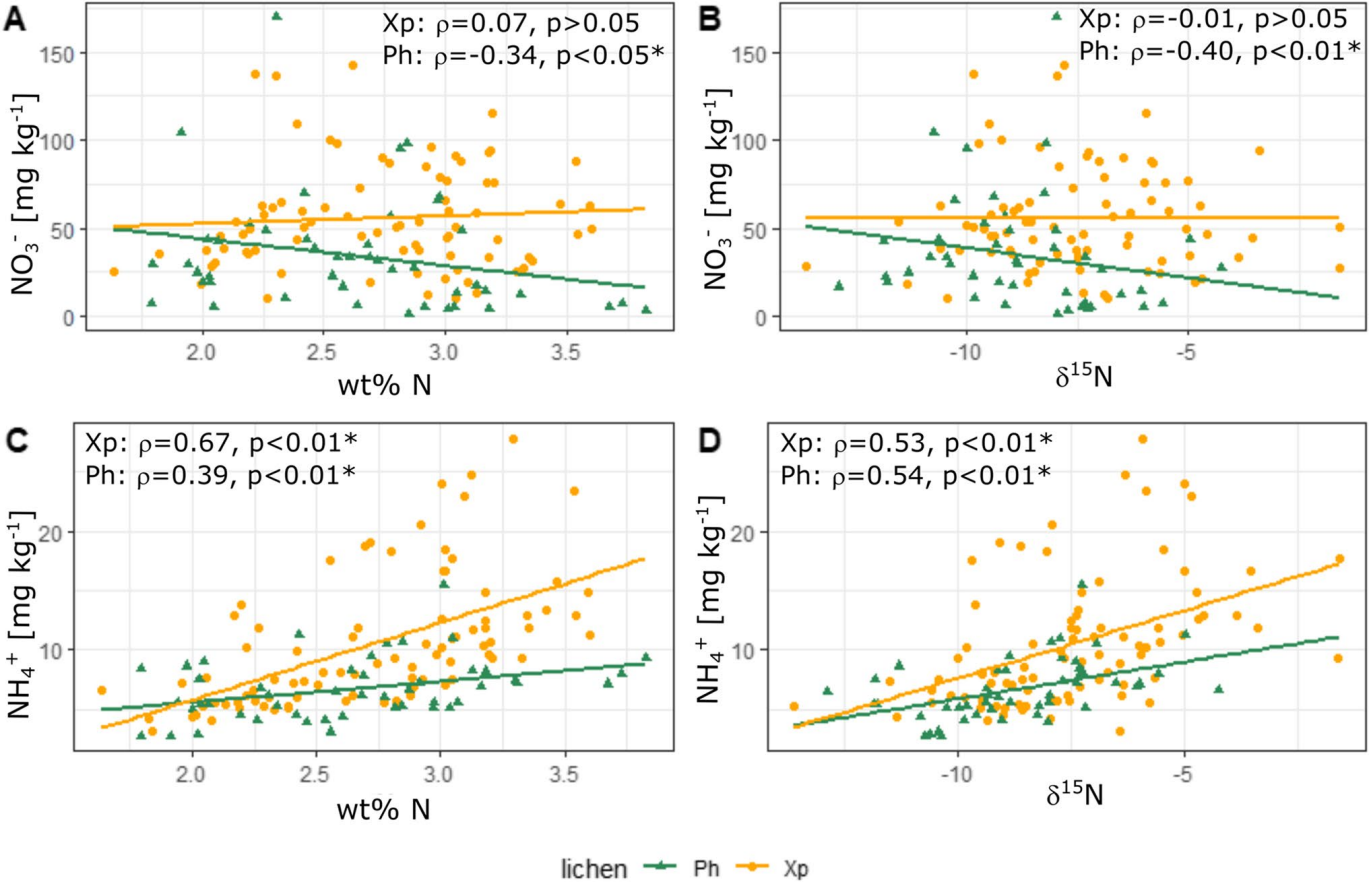
Scatterplots of nitrate (A and B) and ammonium (C and D) concentrations for comparison with (A and C) nitrogen contents [wt%] and (B and D) δ^15^N [‰] in *X. parietina* (Xp) and *Physcia* spp. (Ph; analytical precision, lichen CRM BCR-482-derived for N wt% ± 0.04; δ^15^N ± 0.23; NO_3_^−^ ± 0.45 and NH_4_.^+^  ± 0.25: not shown in figure); displayed with correlation statistics (Spearman ρ, statistically significant correlation marked with *)

To further investigate potential impacts by nitrogen compounds on lichens, as reported by Pinho et al. (2017), N wt% and δ^15^N was linked to lichen NO_3_^−^ and NH_4_^+^ (Fig. 7), using the nitrogen speciation method described by Niepsch et al. (2021b). Species-specific responses to nitrogen compounds, particularly for NO_3_^−^ were recorded, with *Physcia* spp. wt% N and δ^15^N being statistically significant correlated with NO_3_^−^ concentrations, whereas no such relationship was recorded for *X. parietina* (Fig. 7). In lichens, the mycobiont is responsible for nitrate assimilation, which has been found to be less effective compared to ammonium assimilation (Dahlman et al. 2004; Gaio-Oliveira et al. 2005b; Hauck 2010; Palmqvist and Dahlman 2006; Pavlova and Maslov 2008). Such an inter-species difference should be considered, when applying a lichen biomonitoring approach and comparing (urban) environments.

On the contrary, it is evident that NH_4_^+^, compared to NO_3_^−^, has a significantly positive impact on lichen wt% N and δ^15^N values. Elevated levels of ammonia/ammonium have been reported in urban areas, due to three-way catalyst cars being connected to increases in airborne ammonia and subsequently NH_4_^+^ (Bishop and Stedman 2015; Cape et al. 2004; Sun et al. 2017). Comparably, NH_3_ from vehicular emissions and industrial sources (Fig. 7) were found in more positive ranges, compared to highly negative ranges of agricultural nitrogen compound isotopic signatures (i.e. pig farm and livestock; Fig. 6, that may have impacted on recorded lichen δ^15^N. Additionally, NH_4_^+^ from precipitation (Fig. 6) could have impacted on recorded lichen δ^15^N values. For instance, Xu et al. (2021) reported that lichen δ^15^N lower than -7.8‰ indicate a dominance of ^15^N-depleted NH_4_^+^ relative to ^15^N-enriched NO_3_^−^ in wet deposition. Moreover, they reported a higher contribution from volatilization NH_3_ (e.g. from waste and fertilizers) than combustion NH_3_ (e.g. from vehicular emissions, coal combustion and biomass burning) to *X. parietina* δ^15^N in the Beijing-Tianjin-Hebei region, China. However, δ^15^N values from lichen samples across Manchester suggest local sources (e.g. vehicular emissions) of nitrogen pollution, although not allowing for a clear distinction of particular pollution sources. Hence, a more complex influence of NO_x_ and NH_x_ compounds across Manchester is suggested.

Nonetheless, lichen wt% N and δ^15^N stable-isotope-ratio signatures can provide beneficial information on spatial variability of nitrogen compounds (ii), particularly at locations that are not regularly covered by automated air quality monitoring stations. Moreover, lichen NO_3_^−^ and NH_4_^+^ concentrations, when combined with wt% N and δ^15^N allow for finer detail of air quality and aid identification of areas with poor air quality and potential increased human health risks (iii). To further pinpoint vehicular-related influences, traffic marker metal (e.g., Pb and Zn) concentrations in lichens could be combined with δ^15^N signatures (Bermejo-Orduna et al. 2014; Gerdol et al. 2002; Pearson et al. 2000).

#### Lichen sulfur stable-isotope ratios (δ^34^S)

**Fig. 8:**
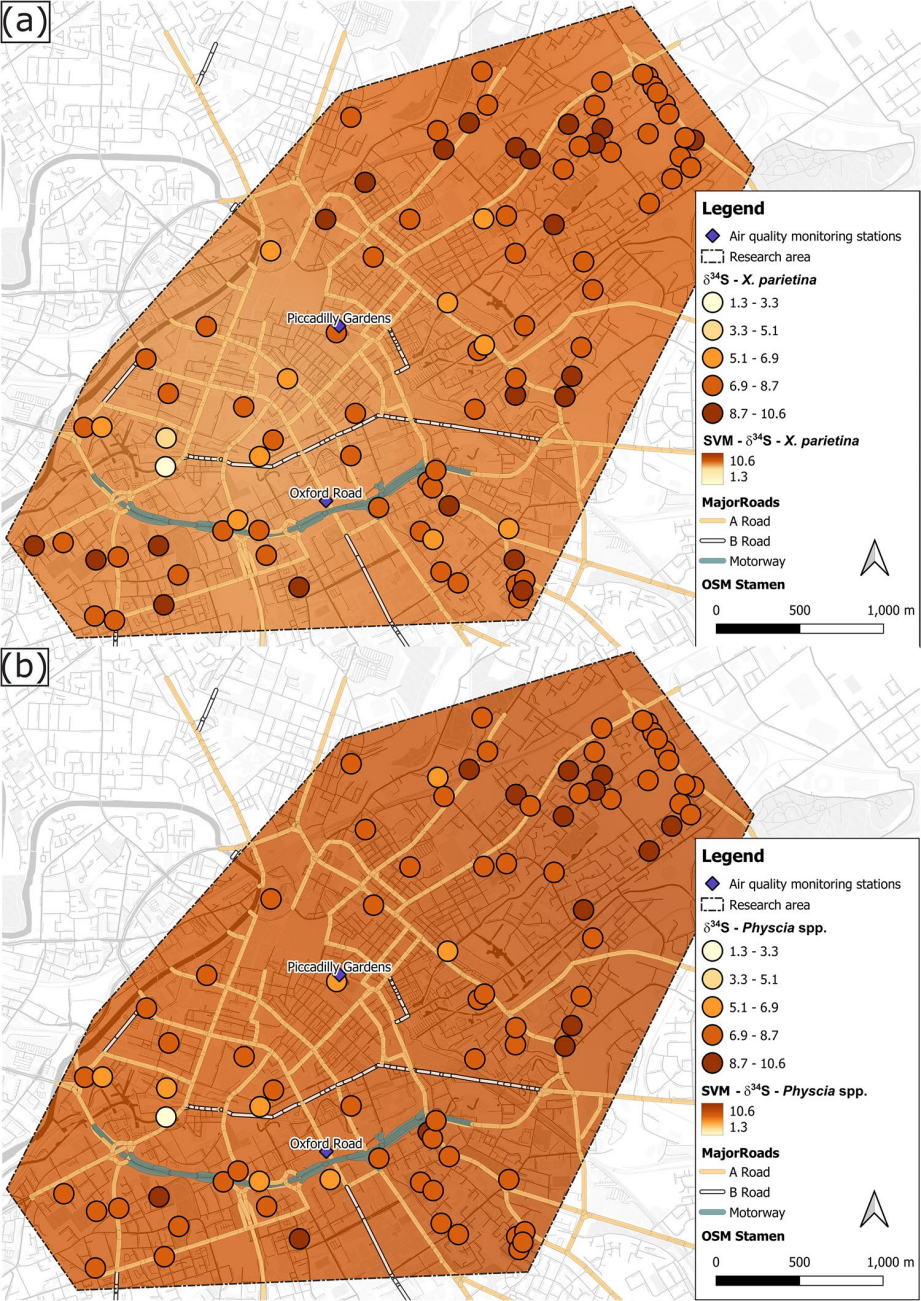
δ^34^S values (‰) of (**a**) *X. parietina* and (**b**) *Physcia* spp. colour-coded by ^34^S enrichment (more positive values) across the City of Manchester; displayed with automated monitoring stations and major road network (A- and B-road and motorway); displayed with SVM interpolated values (same colouring and range) including Manchester’s urban factors

Across Manchester δ^34^S values in *X. parietina* and *Physcia* spp. ranged between 1.3 and 10.3‰ and 1.5 and 9.6‰, respectively. Studies reported that δ_34_S-values < 10‰ are related to anthropogenic sulfur sources (Wadleigh 2003; Wadleigh and Blake 1999; Wiseman and Wadleigh 2002). Moreover, low δ^34^S values (< 10‰), together with increases in wt% S, have been associated with anthropogenic sulfur sources (Wadleigh and Blake 1999), as is evident for both lichens utilised in this study (*X. parietina* and *Physcia* spp.; Fig. 6 f), i.e. a statistically significant negative correlation (*X. parietina*: *p* < 0.01, *Physcia spp*: *p* < 0.05) was recorded for δ^34^S and wt% S. In this study, *X. parietina* samples were more enriched in ^34^S in more residential surroundings in the northeast and southwest of the research area, whereas lower δ^34^S values were recorded in the city centre area (Fig. 8), i.e. where higher traffic counts and surrounding building heights (Table 2) suggest localised influences. For instance, sulfur-containing compounds and particulates, e.g. from residential combustion (using petroleum coke; NAEI 2018b) and vehicular emissions (AQEG 2012; Clean Air Greater Manchester 2021) may have impacted on lichen stable-isotope-ratio signatures.

**Fig. 9:**
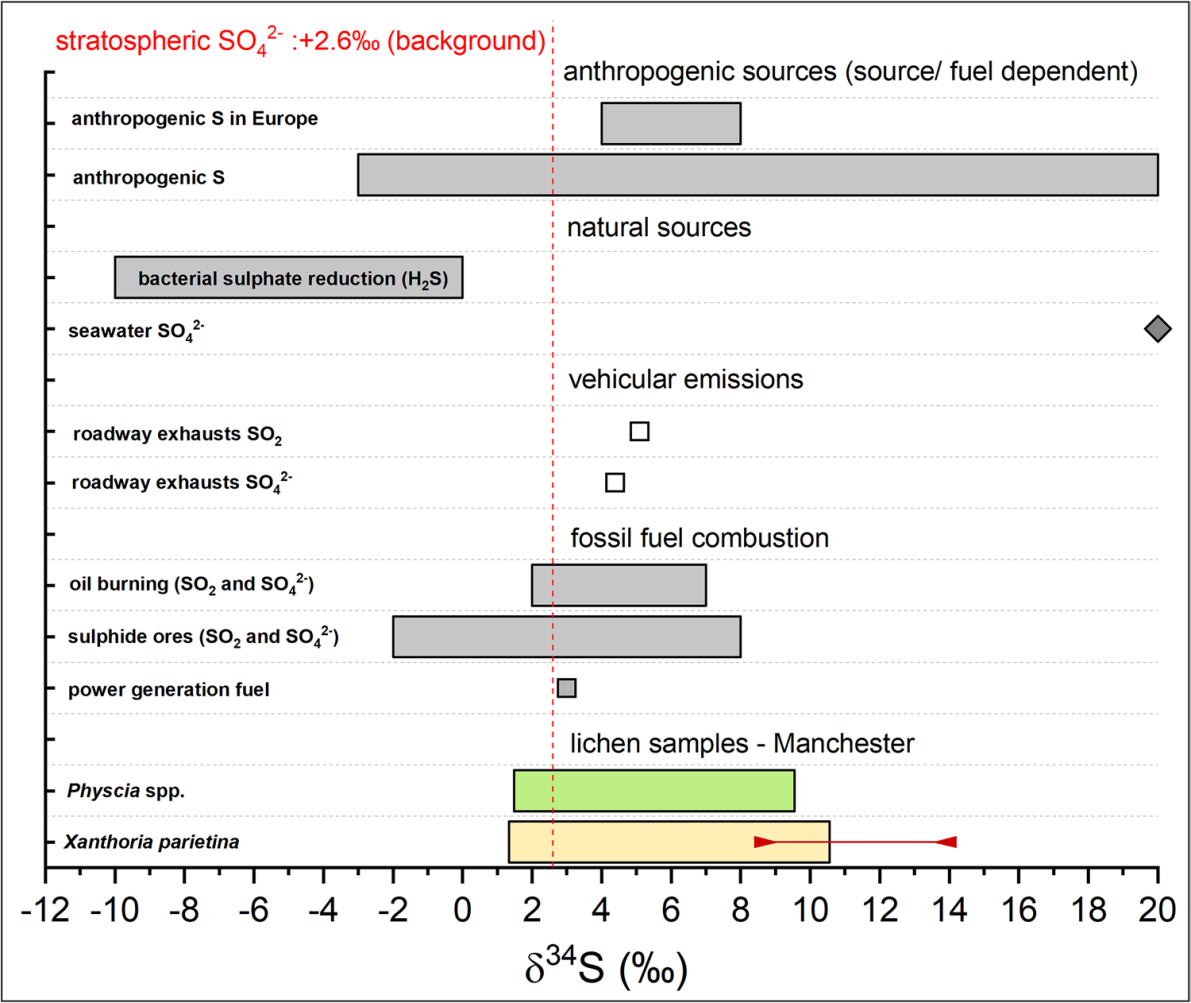
δ^34^S values of potential atmospheric pollutants and pollution sources reported in the literature (Case and Krouse 1980; Cortecci and Longinelli 1970; Norman 2004; Wadleigh 2003; Wadleigh and Blake 1999; Wiseman and Wadleigh 2002) to undertake lichen δ^34^S value source apportionment; displayed with δ-values of lichens (*X. parietina* and *Physcia* spp.) sampled across Manchester (rural *X. parietina* are displayed as red solid line on bar for comparison)

Sulfur enters the atmosphere from different sources and SO_2_ can be widely distributed (Pinho et al. 2008). However, lichen sulfur-isotope-ratio signatures are related to their proximal atmosphere, and little stable-isotope fractionation during assimilatory processes has been demonstrated, allowing the use of δ^34^S values to fingerprint natural versus anthropogenic sulfur sources (Batts et al. 2004; Wadleigh 2003; Wadleigh and Blake 1999; Wiseman and Wadleigh 2002). Manchester lichen δ^34^S ranges are in ranges of anthropogenic sources, e.g. fuel combustion and vehicular emissions (Fig. 9), whereas natural sources (e.g. bacterial H_2_S and seawater SO_4_^2−^) δ^34^S are in highly negative and positive ranges, respectively. Additionally, low recorded atmospheric SO_2_ concentrations (at Manchester Piccadilly) and an annual reduction of approximately 5% (each year) between 2000 and 2017 (Sicard et al. 2021), suggest that lichen sulfur-isotope-ratio signatures are likely to represent the anthropogenic sources and “local” sulfur concentrations for the urban environment of Manchester (Spiro et al. 2002; Wadleigh 2003; Wadleigh and Blake 1999; Wiseman and Wadleigh 2002). Thus, this study’s findings support the use of lichen wt% S and δ^34^S signatures to evaluate spatial variability in airborne sulfur pollution, and potentially fingerprint potential sources in an urban environment (ii).

Meteorological conditions (e.g., wind dispersion and wash-off effects by precipitation; Spiro et al. 2002) as well other sulfur compounds (e.g., organosulfur from biological process; Perraud et al. 2015) might have impacted on lichen δ^34^S values, but were beyond the scope of this work. Detailed characterisation of lichen δ^34^S values directly proximal to potential sulfur sources, as well as analysis of different fuel types used in energy production and residential heating across Manchester, would improve further source apportionment using δ^34^S.

### Comparison of CNS contents and stable-isotope ratios in urban and rural *X. parietina* samples and the implications for lichen biomonitoring studies

*X. parietina* samples (*N* = 12; Fig. S3) were used to compare the contrasting urban and rural environments and to evaluate specific differences in CNS contents and stable-isotope-ratio signatures (individual rural lichen wt% CNS and δ-values in Table S7). Overall, wt% C and wt% N were higher and significantly (*p* < 0.01) different in rural *X. parietina* lichens, compared to their urban counterparts (Fig. S8 a and b), whereas wt% S contents were lower in rural *X. parietina* samples (Fig. S8 c). Comparison of stable-isotope-ratio signatures (δ^13^C, δ^15^N and δ^34^S), showed significant (*p* < 0.01) differences between rural and urban *X. parietina*, with enrichment in ^13^C, ^15^N and ^34^S in rural lichens (Fig. S8 d, e and f).

For wt% C and δ^13^C (Fig. S8 a and d), recorded differences are most likely linked to environmental conditions in the analysed environment (i.e. urban and rural) and intra- and inter-variability of carbon contents in lichens (Beck and Mayr 2012; Johansson et al. 2010; Máguas et al. 2013). Further, ranges of (organic) carbon contents (% dry weight) in poultry manure (from different poultry in the UK) were reported between 24.2–34.8% (Nicholson et al. 1996), suggesting potential deposition related influences on lichens proximal to the poultry farm.

In contrast, elevated wt% N in rural *X. parietina* (Fig. S8 b) is in accordance with lichen studies undertaken in agricultural surroundings and livestock areas (Boltersdorf and Werner 2013; Franzen-Reuter 2004; Frati et al. 2007), being linked to nitrogen emissions from poultry manure and nitrogen-containing fertilizers (AQEG 2018; Nahm 2005). Moreover, wt% N decreased with increased distance from the poultry farm (r = -0.61; *p* < 0.05), suggesting increased availability of nutrients (e.g. NO_x_ and NH_x_), increased lichen biomass, increased chlorophyll content and subsequent increased photosynthetic capacity, as well as net gain from CO_2_ assimilation, and thus potentially explaining elevated wt% C in rural *X. parietina* (Dahlman et al. 2002; Gaio-Oliveira et al. 2005b; Hauck 2010; Johansson et al. 2010; Nybakken et al. 2009). Interestingly, rural lichen δ^15^N values (Fig. S8 e) were more positive than urban samples, with δ^15^N particularly enriched in close proximity to the poultry farm. More negative δ^15^N was expected in the agricultural area, compared to Manchester, due to greater contribution of reduced nitrogen, i.e. NH_x_ (Boltersdorf et al. 2014; Pearson et al. 2000; Sutton et al. 2004). However, poultry farm emissions might also be isotopically different compared to other livestock; e.g., poultry farm emitted NO_2_ has been reported to have a δ^15^N value of -8.5‰ (Felix and Elliott 2014; Liu et al. 2016) that could explain enrichment in ^15^N in the rural *X. parietina* analysed in this study. Consequently, comparison of lichen δ^15^N in the analysed urban (Manchester) and rural (poultry farm) environments has not allowed for a clear distinction of NO_x_ (i.e. urban) and/or NH_x_-related (i.e. rural) influences and additional research on the δ^15^N values of NO_x_ and NH_x_ compounds (and their impact on lichen stable-isotope ratios) in these environments is required.

Differences in wt% S between rural and urban lichen sampled (Fig. S8 c) are most likely related to lower SO_2_ concentrations in the rural environment. For instance, SO_2_ emissions in the UK from agriculture accounted for small amounts (11.58 kilotons in 2016) of total emissions, compared to anthropogenic sources (168 kilotons in 2016) (NAEI 2018b). Additionally, δ^34^S in urban samples were less positive, compared to rural samples (Fig. S8 d) and δ^34^S did not decrease with increasing wt% S in rural samples, suggesting minor anthropogenic influences. Hence, δ^34^S (as well as wt% S) in rural lichen samples may well simply record the ‘background/natural’ sulfur concentrations in the rural environment, which is less affected by anthropogenic sources. Hence, the applied lichen biomonitoring approach was considered a useful tool to assess and compare these contrasting environments for (anthropogenic) sources of sulfur compounds.

In general, lichen wt% N and wt% S allow for comparison of atmospheric pollution in contrasting urban and rural environments; e.g., poultry farm and agricultural emissions for rural lichen wt% N and combustion sources for urban lichen wt% S. However, lichen δ^13^C and δ^15^N values suggest more complex airborne pollutant influences on lichens, with δ^34^S solely allowing for differentiation of anthropogenic impacts in these contrasting environments. However, a lichen biomonitoring approach facilitates a finer spatial detail of air quality assessment and can help to identify urban areas where improvements to air quality are required, thereby further supporting existing automated air quality measurements. Nonetheless, *X. parietina* samples, obtained around a poultry farm might not represent optimal sites for comparison with urban environments, when using CNS contents and stable-isotope-ratio signatures, because agricultural influences, particularly for nitrogen compounds, may have impacted on recorded wt% N and δ^15^N values that warrant further investigation. To further improve the comparison between environments and fingerprinting of potential sources, more pristine (if possible) remote lichen samples (e.g. sampled from areas with lower concentrations of atmospheric pollutants) are suggested. Additional environmental compartments, e.g. soil and atmospheric compound wt% CNS their associated stable-isotope-ratio compositions would provide additional information and aid contribution of these compounds to lichen pollutant loadings and isotopic compositions.

## Conclusion

In this study two lichens (*X. parietina* and *Physcia* spp.) were used as biomonitors to identify high-resolution spatial variability of air quality, and also possible pollution source apportionment, across the Manchester (UK) urban environment. Spatial variability of airborne nitrogen and sulfur compounds across Manchester was identified using these lichens, whereas identification of deteriorated air quality by carbon compounds was not completely possible (i.e., due to temporal change superimposed on spatial variability of lichen signals).

Central Manchester’s complex urban layout has impacted on recorded lichen carbon, nitrogen and sulfur loadings and associated stable-isotope-ratio signatures, allowing for identification of areas of deteriorated air quality, and thus human health concern, where ameliorative actions should be targeted. Species-specific differences in lichen chemistry were recorded and such variability ought to be considered when using lichens as biomonitors of spatial variability of airborne pollution; use of a ‘single’ lichen species is suggested, particularly when studying urban environments.

Source apportionment of airborne pollution in Manchester was not possible from lichen δ^13^C and δ^15^N values, but a more complex impact of nitrogen compounds (NO_x_ and NH_x_) was found that warrants further investigation, i.e. using lichen NO_3_^−^ and NH_4_^+^ concentrations. In contrast, combined Manchester lichen wt% S and δ^34^S values can primarily be attributed to anthropogenic sources of sulfur, such as from domestic heating emissions. Additional research on stable-isotope-ratio signatures of possible anthropogenic sources, e.g. particulates and gaseous compounds (Berner and Felix 2020), would significantly improve source apportionment studies.

This study contributes to further development of an easy-to-use, relatively quick, and low-cost lichen biomonitoring approach, applicable to the assessment of urban airborne pollutants at a finer spatial detail which is transferable to comparable urban areas. Such an approach can be undertaken in areas without automated air quality measurements, or lichen biomonitoring of this type can support, and be ground-truthed, by automated monitoring programmes. A lichen biomonitoring approach using wt% N and S wt% with their associated stable-isotope ratios, can provide a promising tool for local authorities, stakeholders and NGOs to identify ‘hotspots’ of poor air quality and areas of human health concern. Thereby, supporting development of potential airborne pollution amelioration and mitigation strategies, e.g. emission reductions, pollution taxes and ‘clean air zones’.

## Supplementary Information

Below is the link to the electronic supplementary material.

Supplementary file1 (DOCX 7381 KB)

## Data Availability

All data generated or analysed during this study are included in the article and its supplementary information.
