# Supplementary material for: High spatial resolution assessment of air quality in urban centres using lichen carbon, nitrogen and sulfur contents and stable-isotope-ratio signatures
Source: Environ Sci Pollut Res Int. 2023 Mar 30;30(20):58731–54. doi: 10.1007/s11356-023-26652-8 (PMC10163116; doi:10.1007/s11356-023-26652-8)
Supplement: Supplementary file 1 — Supplementary file1 (DOCX 7381 KB) [file 11356_2023_26652_MOESM1_ESM.docx]

High spatial resolution assessment of air quality in urban centres using lichen carbon, nitrogen and sulfur contents and stable-isotope-ratio signatures

Daniel Niepsch^1^, Leon J. Clarke^1^, Jason Newton^2^_,_ Konstantinos Tzoulas^1^ and Gina Cavan^1^

^1^ Department of Natural Sciences, Faculty of Science and Engineering, Manchester Metropolitan University, M1 5GD, Manchester, UK

^2^ Stable Isotope Ecology Laboratory, Scottish Universities Environmental Research Centre (SUERC), G75 0QF, East Kilbride, UK

Corresponding author: [Daniel.Niepsch@gmx.de](mailto:Daniel.Niepsch@gmx.de)

*Supplementary Material*


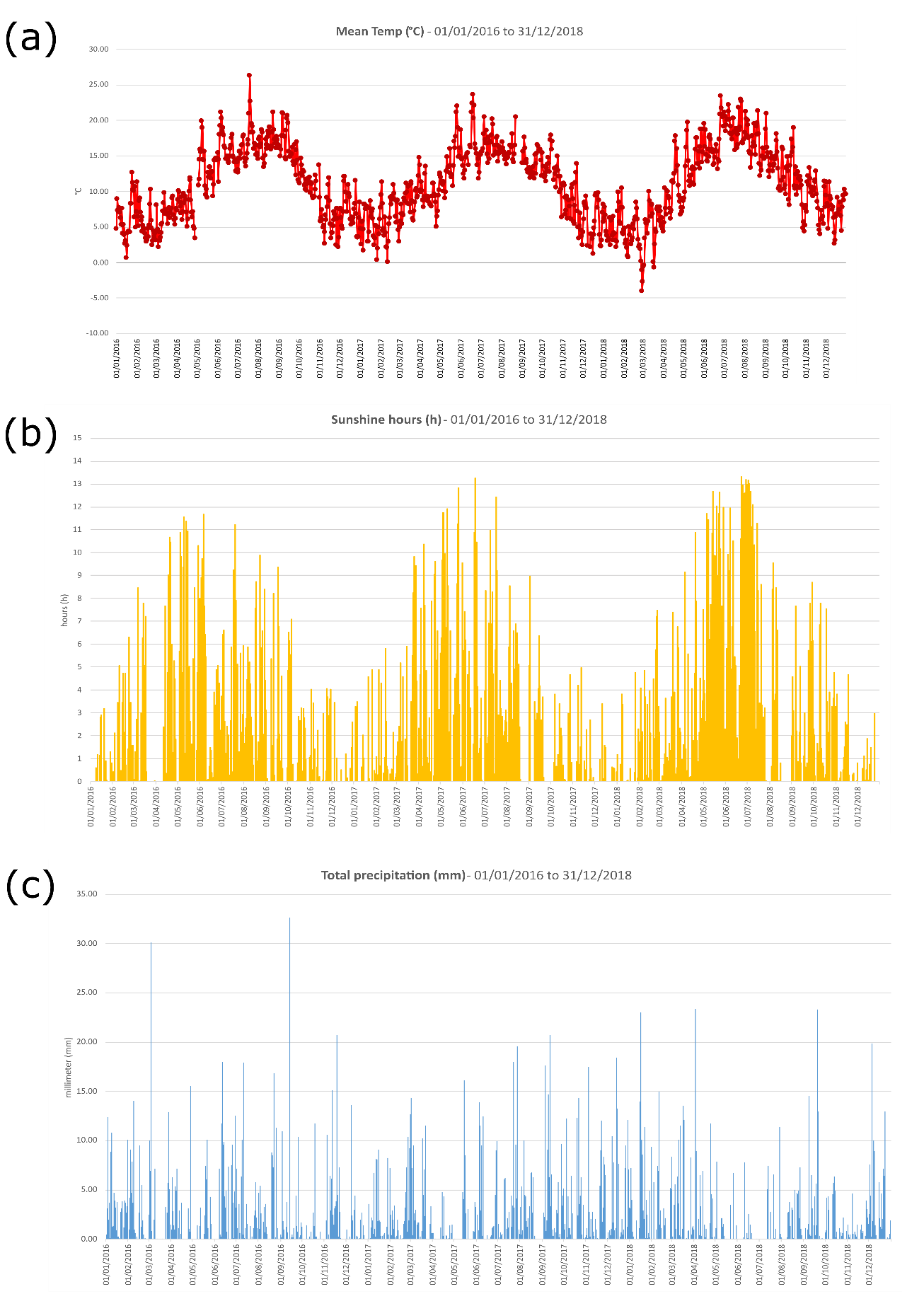


**Fig. S1:** Climatic data for (a) mean temperature (°C), (b) sunshine hours (h) and (c) total precipitation for the period 01/01/2016 to 31/12/2018, which incorporates this study’s sampling period. Data obtained from the ‘Whitworth Meteorological Observatory’, Longitude: N53.467374, Latitude: W2.232006, Altitude: 43 m; “Whitworth Meterological Observatory - Data Archive,” 2018).


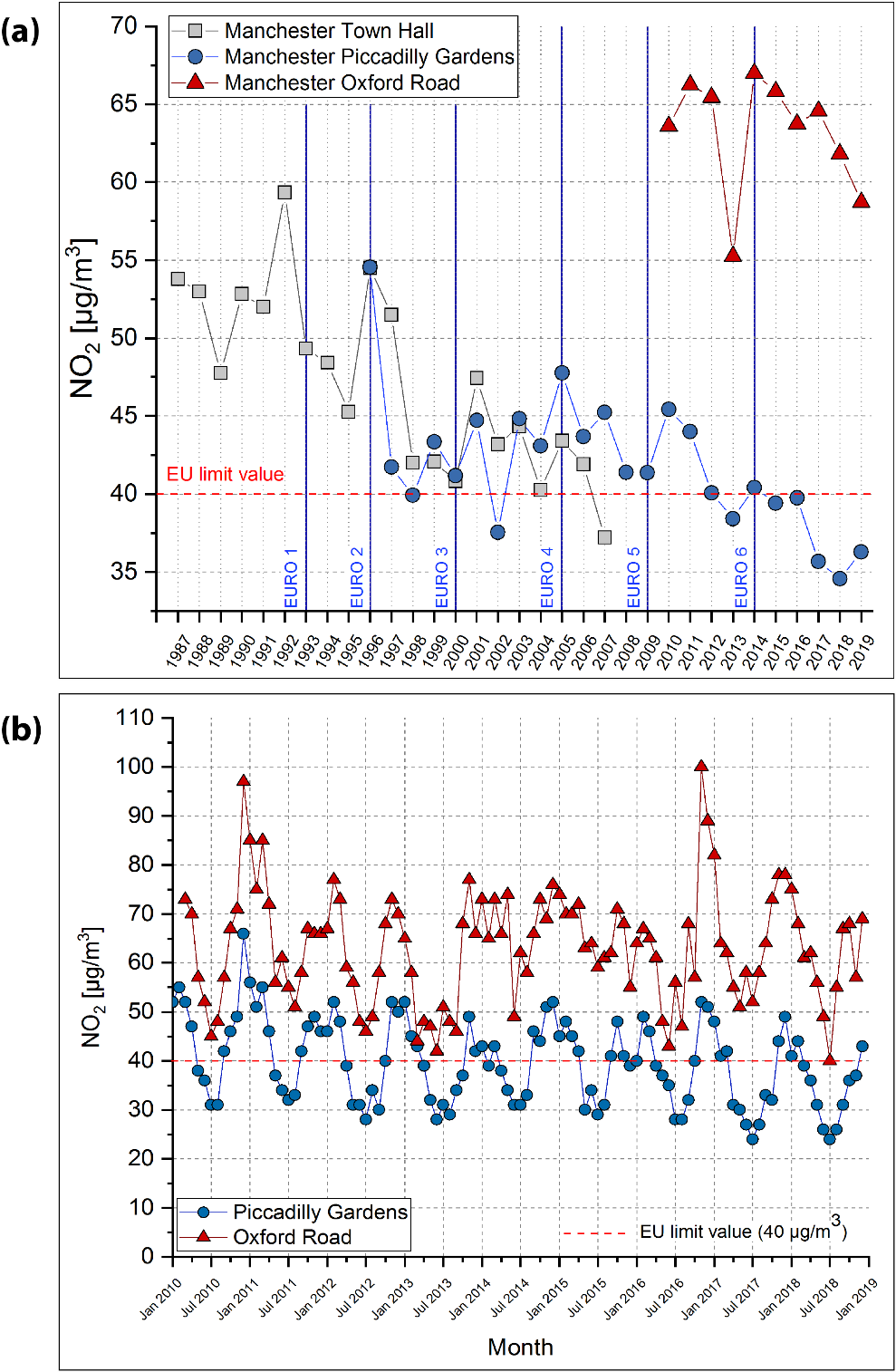


**Fig. S2:** (a) NO_2_ concentrations [µg m^-3^] annual mean between 1987 and 2019 measured at Manchester automated monitoring stations (including Town Hall monitoring station – terminated in 2007), with introduction dates of EURO emission standards and (b) monthly mean at the automated monitoring stations (Piccadilly Gardens: blue and Oxford Road: red) for the years 2010 to 2019 (Air Quality England, 2018a, 2018c)


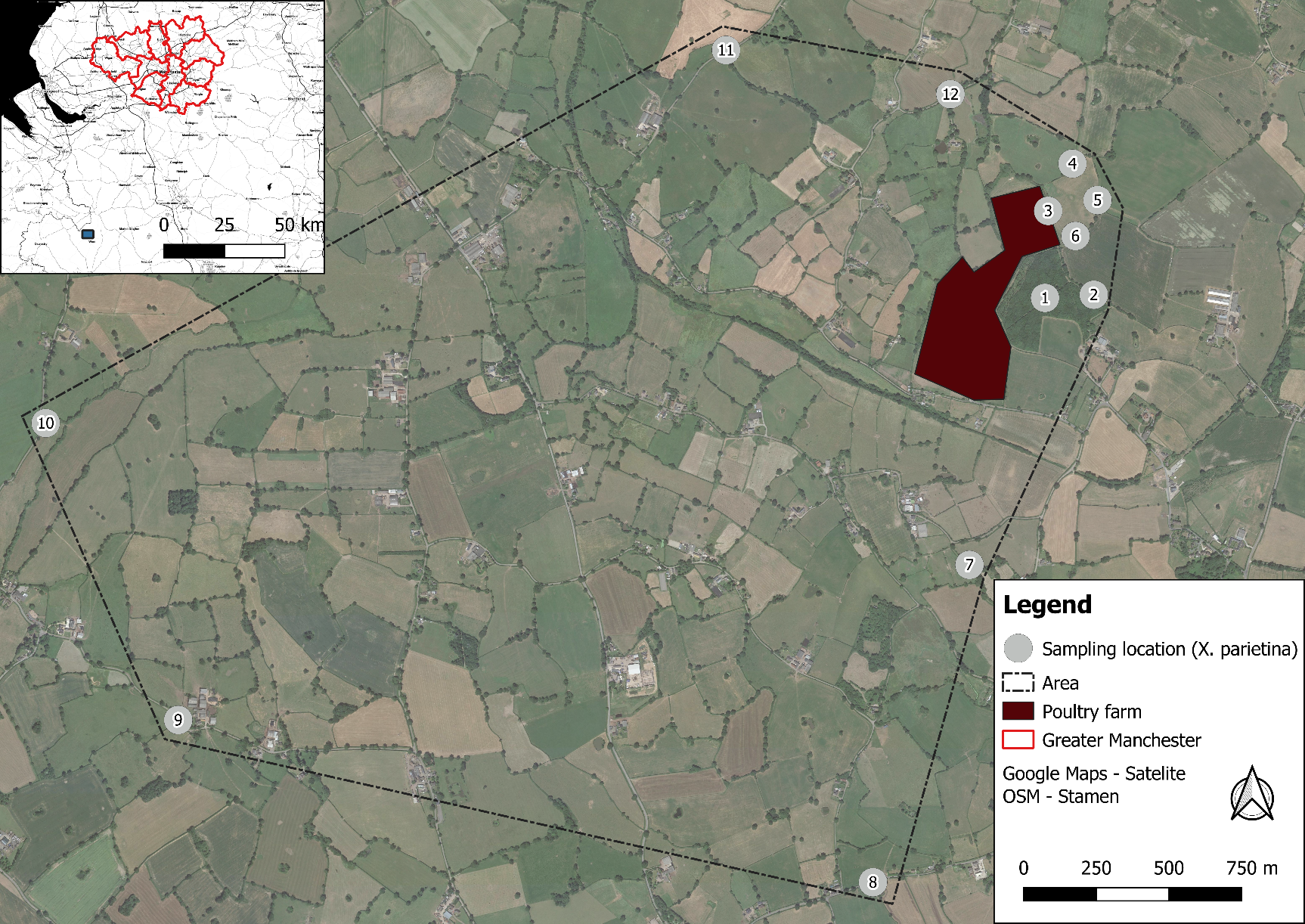


**Fig. S3:** Rural lichen sampling sites (*X. parietina*; N=12) distributed around a poultry farm in Shrewsbury, UK (sampled in May 2018); displayed with location of poultry farm in relation to Greater Manchester (upper left corner, blue square); background map: Google Maps - Satellite (map data 2018 - Google)

**Tab. S1:** Analysed carbon, nitrogen and sulphur contents (wt%) of lichen CRM (No. 482) by CN (N=31) analyser and IRMS (N=43) and certified values, displayed with ± 1x standard deviation (Quevauviller et al., 1996) and LECO TrueSpec reference material ‘Rice Flour’ (502-278, N=32) for CN analysis (presented as ± 1x standard deviation; N/A – not measured with CN analyser or IRMS

|  | CN analyser | IRMS | Certified Value |  | Accuracy (%)  CN analyser | Accuracy (%) IRMS |  | Precision (%CV) CN analyser | Precision (%CV) IRMS |  |
| --- | --- | --- | --- | --- | --- | --- | --- | --- | --- | --- |
| Nitrogen (N wt%) | 1.78 ± 0.04 | 1.87 ± 0.15 | 1.743 ± 0.025 |  | 102 | 107 |  | 2.19 | 8.3 |  |
| Carbon (C wt%) | 44.05 ± 0.55 | 45.48 ± 5.16 | 44.7 ± 0.700 |  | 99 | 102 |  | 1.25 | 11.3 |  |
| Sulphur (S wt%) | N/A | 0.18 ± 0.02 | 0.2166 ± 0.029 |  | N/A | 85 |  | N/A | 13.5 |  |
| LECO reference material – rice flour (CN analysis) | | | | | | | | | | |
| Nitrogen (N wt%) | 0.99 ± 0.04 | N/A | 1.13 |  | 88 | N/A |  | 3.85 | N/A |  |
| Carbon (C wt%) | 40.95 ± 0.41 | N/A | 44.46 |  | 92 | N/A |  | 1.00 | N/A |  |

**Tab. S2:** Measured and accepted isotope ratios (mean ± 1x standard deviation) for international isotope standards (USGS40, IAEA-S1 to S3, MSAG2, M2 and SAAG2) and lichen CRM (N=43) used during IRMS analysis (NA = not applicable, no values available); displayed with overall accuracy (%) and precision (%CV).

| Reference Material | Measured Values | | | Accepted Values | | | Accuracy (%) | | | Precision (%CV) | | |
| --- | --- | --- | --- | --- | --- | --- | --- | --- | --- | --- | --- | --- |
|  | δ^15^N | δ^13^C | δ^34^S | δ^15^N | δ^13^C | δ^34^S | δ^15^N | δ^13^C | δ^34^S | δ^15^N | δ^13^C | δ^34^S |
| USGS40  (N=8) | -4.61 ±0.11 | -26.32 ± 0.09 | NA | -4.52 ± 0.06 | -26.39 ± 0.04 | NA | 102 | 99.7 | N/A | 2.39 | 0.34 | N/A |
| IAEA-S2  (N=11) | NA | NA | 22.23 ±0.35 | NA | NA | 22.62 ± 0.20 | N/A | N/A | 98.3 | N/A | N/A | 1.58 |
| IAEA-S3  (N=11) | NA | NA | -32.44 ±0.26 | NA | NA | -32.49 ± 0.20 | N/A | N/A | 99.9 | N/A | N/A | 0.80 |
| MSAG2  (N=121) | 2.28 ±0.03 | -21.30 ±0.03 | 6.27 ±0.13 | 2.24 ±0.09 | -21.23 ±0.12 | 6.18 ± 0.43 | 100.8 | 99.8 | 101.6 | 1.14 | 0.13 | 2.09 |
| M2  (N=78) | 32.70 ±0.002 | -34.21 ±0.01 | 14.23 ±0.39 | 32.70 ±0.27 | -34.28 ± 0.11 | 14.43 ± 0.46 | 100 | 100.1 | 101.3 | 0.06 | 0.04 | 2.7 |
| Lichen CRM 482 | -7.95 ± 0.23 | -25.04 ± 0.61 | 5.58 ± 0.62 | NA | NA | NA | N/A | N/A | N/A | 2.9 | 2.4 | 11.0 |

**Tab. S3:** Urban influencing factors, data source and data classification justification, based on pollutant dispersion and human health studies, used for Geographically Weighed Regression (GWR) of lichen CNS contents (wt%) and stable-isotope ratio signatures (δ-values) for high-spatial resolution analysis of air quality in Manchester; with used datasets and data sources, classification and justification of data classification

| Urban influencing factor | Dataset used and source | Data classification | Justification |
| --- | --- | --- | --- |
| Major road classes | Major road classes – A-, B-roads and motorways (Digimap - Ordnance Survey, 2016) | **M** – motorway  **A** – A-Road  **B** – B-road | Decline of NO_x_ and NO_2_ from major roads within the first 200 m (Bermejo-Orduna et al., 2014; Gombert et al., 2003; Laffray et al., 2010) |
| Distance to Major Road | Categorised by linear buffer (25, 50, 100 and 200m) superimposed on road network | **1:** <25 m  **2:** 25 to 50m  **3:** 50 to 100m  **4:** 100 to 200 m  **5:** >200m |  |
| Traffic counts | Annual Average Daily Traffic flow (AADF, 2017) – all vehicles (DfT, 2017) | **1:** <10.000  **2:** 10.000 to 20.000  **3:** 20.000 to 30.000  **4:** >30.000 | Dynamic traffic movement from measured/estimated data – available as point data only; total vehicles per day used |
| Building heights | OS Building Heights, ‘relative height from ground level to highest part of rood (relHmax)’ (Digimap - Ordnance Survey, 2017); mean building height in 50m buffer of sampling site | **1:** <10m  **2:** 10 to 20m  **3:** >20m | High urban density affect flow patterns (i.e. ventilation), causing poor air and impacting on pedestrian health (Britter and Hanna, 2003; Buccolieri et al., 2010; Hertel and Goodsite, 2009; Lo and Ngan, 2015; Shen et al., 2017) |
| Distance to green space | OS Open green space, distance measured within GIS software, ‘amount of greenspace’ (area in m^2^) measured using 50m buffer around sampling site (Digimap - Ordnance Survey, 2018) | **1:** <100m  **2:** 100m to 200m  **3:** 200m to 300m  **4:** 300m to 400m  **5:** 400m to 500m  **6:** >500m | Human health studies reporting decreased impacts of air pollution within the first 30m and 500m of greenspaces, positive impact of vegetation on air quality and air pollution (Browning and Lee, 2017; Dadvand et al., 2012a, 2012b; Janhäll, 2015; Salmond et al., 2013) |


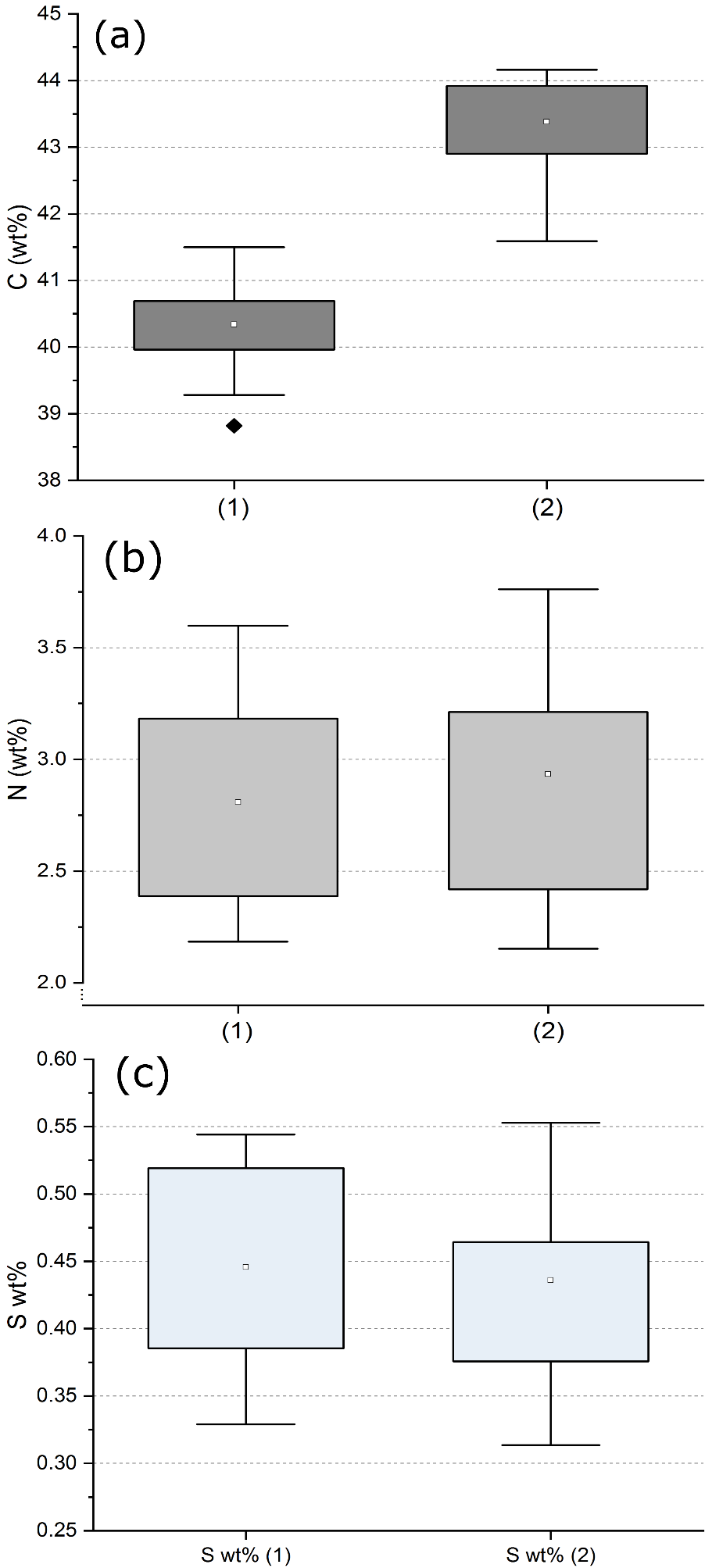


**Fig. S4:** Box-Whisker plots (25th to 75th percentile; displayed with mean as white square) for lichen carbon (a), nitrogen (b) and sulfur (c) contents (in wt%) for sampling periods in 2016/2017 (1) and revisited in 2018 (2)

**Tab. S4:** Individual CNS contents (wt%) and stable-isotope ratio signatures (‰), recorded in *X. parietina* (N=94) and *Physcia* spp. (N=86), sampled across Manchester; with XY-coordinates (OSGB1936) and site ID, sampling locations with site-ID shown in **Figure S4;** N/A – not enough lichen material obtained for analysis

| Site-ID | X  (OSGB 1936) | Y  (OSGB 1936) | *X. parietina* | | | | | | *Physcia* spp. | | | | | |
| --- | --- | --- | --- | --- | --- | --- | --- | --- | --- | --- | --- | --- | --- | --- |
|  |  |  | C wt% | δ^13^C | N wt% | δ^15^N | S wt% | δ^34^S | C wt% | δ^13^C | N wt% | δ^15^N | S wt% | δ^34^S |
| 1 | 385143 | 398190 | 40.70 | -26.09 | 2.50 | -6.83 | 0.382 | 8.45 | 40.93 | -26.49 | 2.48 | -7.39 | 0.249 | 7.72 |
| 2 | 385180 | 398222 | 39.76 | -26.07 | 3.05 | -6.99 | 0.320 | 6.48 | 39.04 | -27.37 | 3.04 | -7.19 | 0.334 | 7.80 |
| 3 | 385420 | 398339 | 39.37 | -24.89 | 2.93 | -6.88 | 0.548 | 8.00 | *N/A* | *N/A* | *N/A* | *N/A* | *N/A* | *N/A* |
| 4 | 385123 | 397837 | 39.90 | -25.22 | 3.04 | -10.43 | 0.489 | 7.29 | 40.83 | -25.35 | 3.00 | -7.75 | 0.379 | 7.57 |
| 5 | 385829 | 398555 | 41.15 | -24.92 | 1.01 | -4.57 | 0.526 | 8.65 | 44.04 | -24.91 | 1.79 | -7.32 | 0.289 | 8.23 |
| 6 | 385760 | 398209 | 40.37 | -24.19 | 3.42 | -5.59 | 0.562 | 7.65 | 41.54 | -25.01 | 3.59 | -6.72 | 0.397 | 7.09 |
| 7 | 385366 | 397922 | 38.82 | -23.36 | 2.04 | -5.65 | 0.530 | 8.85 | 40.82 | -24.41 | 2.58 | -7.27 | 0.346 | 8.11 |
| 8 | 385704 | 398035 | 40.43 | -25.70 | 3.60 | -5.03 | 0.504 | 8.98 | 43.36 | -25.88 | 3.88 | -7.27 | 0.408 | 8.85 |
| 9 | 385662 | 397913 | 40.75 | -25.20 | 2.64 | -7.30 | 0.481 | 8.87 | 40.14 | -24.83 | 2.49 | -8.40 | 0.236 | 9.05 |
| 10 | 385368 | 398023 | 39.28 | -24.03 | 2.82 | -7.53 | 0.462 | 7.99 | 41.28 | -24.33 | 2.94 | -6.02 | 0.341 | 8.52 |
| 11 | 383876 | 396960 | 40.28 | -23.93 | 3.02 | -6.33 | 0.489 | 7.31 | 38.43 | -24.42 | 2.70 | -8.49 | 0.302 | 7.30 |
| 12 | 384072 | 396768 | 40.65 | -24.73 | 1.84 | -6.43 | 0.304 | 9.65 | 44.21 | -25.58 | 1.97 | -6.59 | 0.205 | 9.00 |
| 13 | 383616 | 397107 | 39.54 | -24.58 | 3.23 | -1.58 | 0.501 | 7.85 | 42.30 | -25.34 | 3.47 | -2.03 | 0.389 | 7.37 |
| 14 | 383231 | 397017 | 40.78 | -24.37 | 2.20 | -9.11 | 0.349 | 8.76 | 40.40 | -24.67 | 2.21 | -9.27 | 0.227 | 9.18 |
| 15 | 383705 | 397171 | 39.76 | -26.19 | 3.35 | -1.59 | 0.584 | 6.62 | 43.23 | -25.87 | 3.20 | -3.71 | 0.388 | 7.78 |
| 16 | 385838 | 399661 | 40.72 | -25.10 | 3.08 | -3.54 | 0.462 | 7.54 | 40.27 | -25.44 | 2.87 | -4.24 | 0.297 | 8.05 |
| 17 | 385886 | 399523 | 40.58 | -23.58 | 2.89 | -7.30 | 0.451 | 9.50 | 44.15 | -23.55 | 2.78 | -8.73 | 0.338 | 9.22 |
| 18 | 386160 | 399492 | 40.06 | -24.73 | 2.88 | -6.37 | 0.438 | 8.62 | 41.96 | -24.88 | 2.86 | -7.31 | 0.308 | 8.36 |
| 19 | 386261 | 399667 | 39.32 | -24.49 | 2.00 | -11.35 | 0.350 | 7.02 | 40.22 | -25.58 | 1.81 | -11.40 | 0.228 | 7.01 |
| Tab. S4 continued: Individual CNS contents (wt%) and stable-isotope ratio signatures (‰), recorded in *X. parietina* (N=94) and *Physcia* spp. (N=86), sampled across Manchester; with XY-coordinates (OSGB1936) and site ID, sampling locations with site-ID shown in Figure S4; N/A – not enough lichen material obtained for analysis | | | | | | | | | | | | | | |
| 20 | 385938 | 399381 | 39.52 | -25.12 | 2.50 | -8.41 | 0.538 | 8.09 | 40.79 | -24.45 | 2.76 | -7.55 | 0.346 | 8.11 |
| 21 | 386283 | 399611 | 39.62 | -24.06 | 2.13 | -7.95 | 0.291 | 7.20 | 39.43 | -25.52 | 2.61 | -4.96 | 0.231 | 7.77 |
| 22 | 385166 | 399865 | 40.20 | -25.44 | 2.45 | -11.52 | 0.457 | 8.38 | 42.17 | -24.95 | 2.45 | -12.89 | 0.269 | 7.86 |
| 23 | 385089 | 399557 | 40.06 | -23.99 | 2.67 | -6.70 | 0.446 | 9.42 | 43.78 | -24.53 | 2.91 | -7.56 | 0.338 | 8.90 |
| 24 | 384467 | 399200 | 40.62 | -24.93 | 2.18 | -9.46 | 0.349 | 9.40 | 42.58 | -25.96 | 3.01 | -9.42 | 0.302 | 8.25 |
| 25 | 386434 | 399454 | 40.92 | -24.00 | 3.04 | -7.27 | 0.572 | 9.16 | 41.29 | -24.63 | 3.03 | -7.87 | 0.392 | 8.67 |
| 26 | 384901 | 399510 | 40.42 | -25.70 | 2.89 | -8.53 | 0.492 | 8.45 | 11.73 | -24.50 | 0.80 | -10.75 | 0.051 | 6.25 |
| 27 | 384231 | 398977 | 40.52 | -25.15 | 3.18 | -3.38 | 0.515 | 10.10 | *N/A* | *N/A* | *N/A* | *N/A* | *N/A* | *N/A* |
| 28 | 385359 | 396932 | 39.58 | -24.13 | 3.17 | -6.00 | 0.549 | 9.08 | *N/A* | *N/A* | *N/A* | *N/A* | *N/A* | *N/A* |
| 29 | 385326 | 397120 | 40.81 | -24.35 | 2.56 | -7.37 | 0.411 | 6.75 | 41.06 | -25.42 | 2.51 | -8.02 | 0.277 | 7.76 |
| 30 | 385774 | 398723 | 40.61 | -24.65 | 2.36 | -9.62 | 0.398 | 8.06 | 40.74 | -24.88 | 2.03 | -11.31 | 0.237 | 8.72 |
| 31 | 384382 | 399591 | 40.73 | -23.34 | 2.39 | -8.52 | 0.398 | 8.52 | 41.31 | -24.34 | 2.69 | -6.95 | 0.300 | 7.46 |
| 32 | 384843 | 397402 | 40.92 | -25.77 | 3.00 | -6.88 | 0.548 | 8.34 | 40.35 | -25.13 | 2.80 | -8.47 | 0.291 | 8.90 |
| 33 | 386158 | 399834 | 40.68 | -24.49 | 2.39 | -9.49 | 0.392 | 7.74 | 41.59 | -25.45 | 2.32 | -9.57 | 0.262 | 8.33 |
| 34 | 384869 | 397365 | 40.43 | -24.25 | 3.49 | -5.84 | 0.621 | 7.29 | 42.42 | -24.89 | 3.82 | -5.33 | 0.432 | 7.11 |
| 35 | 385176 | 398981 | 41.56 | -24.95 | 2.62 | -9.14 | 0.451 | 6.78 | 46.12 | -25.66 | 2.74 | -9.00 | 0.326 | 7.34 |
| 36 | 385883 | 399803 | 40.99 | -24.53 | 2.77 | -5.77 | 0.481 | 8.23 | 42.11 | -25.28 | 2.68 | -6.13 | 0.301 | 8.35 |
| 37 | 384798 | 397104 | 39.96 | -23.83 | 2.42 | -9.86 | 0.462 | 7.54 | 40.77 | -23.96 | 2.31 | -11.83 | 0.225 | 8.31 |
| 38 | 386352 | 399353 | 40.04 | -24.39 | 3.04 | -5.96 | 0.535 | 8.07 | 41.36 | -24.89 | 3.71 | -5.56 | 0.404 | 7.55 |
| 39 | 384873 | 397055 | 40.99 | -24.51 | 2.95 | -4.98 | 0.461 | 6.69 | 41.30 | -24.86 | 2.77 | -6.94 | 0.297 | 7.64 |
| 40 | 386190 | 399774 | 40.25 | -23.85 | 2.62 | -7.80 | 0.491 | 7.32 | 41.08 | -24.55 | 3.06 | -7.06 | 0.386 | 7.34 |
| 41 | 386381 | 399468 | 40.64 | -24.55 | 2.37 | -9.46 | 0.456 | 8.43 | 41.83 | -24.12 | 2.71 | -8.28 | 0.321 | 7.23 |
| 42 | 386128 | 399848 | 40.56 | -24.17 | 2.70 | -7.59 | 0.456 | 7.18 | 18.37 | -23.84 | 1.11 | -8.15 | 0.114 | 8.59 |
| Tab. S4 continued: Individual CNS contents (wt%) and stable-isotope ratio signatures (‰), recorded in *X. parietina* (N=94) and *Physcia* spp. (N=86), sampled across Manchester; with XY-coordinates (OSGB1936) and site ID, sampling locations with site-ID shown in Figure S4; N/A – not enough lichen material obtained for analysis | | | | | | | | | | | | | | |
| 43 | 385684 | 399549 | 40.26 | -25.47 | 2.55 | -8.05 | 0.432 | 10.05 | 44.62 | -25.22 | 2.31 | -7.97 | 0.230 | 9.44 |
| 44 | 386302 | 399221 | 40.69 | -23.86 | 2.86 | -9.46 | 0.452 | 8.51 | 41.65 | -24.93 | 2.68 | -10.44 | 0.288 | 8.74 |
| 45 | 385199 | 399664 | 39.61 | -25.24 | 3.02 | -5.45 | 0.537 | 7.86 | 46.65 | -25.46 | 2.82 | -7.75 | 0.306 | 8.36 |
| 46 | 384548 | 397247 | 38.82 | -25.92 | 3.06 | -6.99 | 0.483 | 8.21 | 43.02 | -25.09 | 2.77 | -9.12 | 0.303 | 8.02 |
| 47 | 385369 | 399408 | 40.30 | -22.44 | 2.78 | -8.80 | 0.399 | 9.90 | 41.37 | -23.97 | 2.51 | -8.86 | 0.316 | 8.77 |
| 48 | 384938 | 399397 | 39.63 | -23.49 | 3.03 | -5.84 | 0.564 | 8.97 | 48.40 | -24.59 | 3.90 | -6.26 | 0.442 | 7.84 |
| 49 | 386415 | 399289 | 40.78 | -24.32 | 3.06 | -8.04 | 0.481 | 7.78 | 48.48 | -24.67 | 3.07 | -7.99 | 0.319 | 7.92 |
| 50 | 385373 | 396782 | 40.57 | -24.69 | 2.85 | -9.09 | 0.442 | 7.51 | 48.21 | -25.77 | 2.61 | -10.82 | 0.309 | 8.26 |
| 51 | 386215 | 399738 | 39.41 | -23.63 | 2.22 | -9.83 | 0.421 | 8.06 | 40.08 | -24.65 | 1.88 | -10.74 | 0.278 | 6.92 |
| 52 | 385312 | 398998 | 40.39 | -23.55 | 2.21 | -8.96 | 0.366 | 7.64 | 40.03 | -24.38 | 1.92 | -10.36 | 0.188 | 7.85 |
| 53 | 386167 | 399073 | 39.85 | -24.09 | 2.30 | -9.37 | 0.368 | 8.25 | 40.61 | -25.26 | 2.04 | -10.62 | 0.222 | 8.98 |
| 54 | 385454 | 399341 | 40.57 | -24.25 | 2.23 | -8.66 | 0.376 | 9.70 | 47.03 | -25.11 | 2.40 | -9.03 | 0.294 | 8.14 |
| 55 | 385653 | 399279 | 41.34 | -24.35 | 2.44 | -9.17 | 0.368 | 8.61 | 48.72 | -25.30 | 2.48 | -8.24 | 0.263 | 8.71 |
| 56 | 385597 | 398946 | 39.40 | -24.88 | 2.48 | -10.15 | 0.374 | 8.86 | 46.45 | -25.24 | 2.75 | -9.32 | 0.318 | 8.67 |
| 57 | 384890 | 397470 | 39.93 | -25.41 | 2.61 | -6.44 | 0.455 | 8.57 | 47.23 | -25.53 | 2.84 | -8.20 | 0.345 | 7.70 |
| 58 | 385843 | 399434 | 41.32 | -24.96 | 1.87 | -9.01 | 0.408 | 10.56 | 43.44 | -24.20 | 2.12 | -9.60 | 0.246 | 9.55 |
| 59 | 384969 | 397257 | 40.80 | -24.51 | 2.35 | -8.59 | 0.439 | 8.95 | 46.07 | -24.88 | 2.49 | -8.66 | 0.316 | 8.35 |
| 60 | 385749 | 399415 | 39.97 | -24.05 | 2.41 | -10.60 | 0.422 | 7.24 | 50.14 | -26.04 | 2.22 | -11.79 | 0.301 | 8.00 |
| 61 | 385422 | 396810 | 41.03 | -24.44 | 2.83 | -7.92 | 0.465 | 7.96 | 45.67 | -25.36 | 2.77 | -9.61 | 0.333 | 8.68 |
| 62 | 385024 | 396794 | 40.70 | -24.11 | 2.48 | -8.94 | 0.446 | 8.09 | 46.85 | -24.89 | 2.71 | -9.86 | 0.317 | 8.32 |
| 63 | 384921 | 396860 | 41.50 | -24.39 | 2.56 | -9.20 | 0.419 | 7.61 | 48.24 | -25.46 | 2.82 | -9.99 | 0.305 | 7.70 |
| 64 | 385387 | 396705 | 40.44 | -24.25 | 3.02 | -8.34 | 0.547 | 7.53 | 42.55 | -24.97 | 2.99 | -8.94 | 0.348 | 8.12 |
| Tab. S4 continued: Individual CNS contents (wt%) and stable-isotope ratio signatures (‰), recorded in *X. parietina* (N=94) and *Physcia* spp. (N=86), sampled across Manchester; with XY-coordinates (OSGB1936) and site ID, sampling locations with site-ID shown in Figure S4; N/A – not enough lichen material obtained for analysis | | | | | | | | | | | | | | |
| 65 | 385410 | 396748 | 41.04 | -23.71 | 2.63 | -9.72 | 0.434 | 9.25 | 44.46 | -24.36 | 2.97 | -9.38 | 0.355 | 8.31 |
| 66 | 383156 | 398139 | 41.48 | -24.38 | 3.23 | -6.30 | 0.545 | 7.17 | 47.40 | -25.47 | 3.45 | -7.89 | 0.406 | 7.09 |
| 67 | 383290 | 397932 | 39.83 | -24.38 | 2.71 | -8.61 | 0.516 | 7.74 | 40.52 | -24.65 | 2.18 | -9.81 | 0.282 | 7.65 |
| 68 | 383836 | 397553 | 39.95 | -24.07 | 3.77 | -4.60 | 0.727 | 6.35 | 42.33 | -24.19 | 3.99 | -5.27 | 0.421 | 6.30 |
| 69 | 384734 | 398978 | 40.21 | -26.00 | 3.59 | -4.74 | 0.553 | 7.17 | 46.12 | -25.14 | 3.73 | -5.57 | 0.438 | 7.26 |
| 70 | 383273 | 397492 | 40.99 | -24.74 | 2.81 | -10.01 | 0.654 | 1.34 | 46.03 | -24.86 | 2.97 | -10.26 | 0.408 | 1.48 |
| 71 | 382850 | 396595 | 41.08 | -24.08 | 1.92 | -13.62 | 0.386 | 7.87 | 42.85 | -24.71 | 1.99 | -14.16 | 0.234 | 7.80 |
| 72 | 383902 | 398788 | 40.09 | -25.20 | 3.07 | -7.37 | 0.555 | 6.53 | 43.28 | -26.74 | 3.31 | -8.24 | 0.358 | 6.96 |
| 73 | 383832 | 397110 | 39.63 | -25.06 | 2.95 | -7.45 | 0.493 | 8.05 | 40.41 | -25.40 | 2.92 | -7.37 | 0.404 | 6.72 |
| 74 | 383263 | 396662 | 40.42 | -25.18 | 2.05 | -8.35 | 0.382 | 9.07 | 40.42 | -24.48 | 1.94 | -8.84 | 0.229 | 8.70 |
| 75 | 382857 | 396932 | 41.15 | -23.40 | 2.33 | -8.48 | 0.272 | 9.34 | 49.29 | -24.83 | 3.03 | -8.34 | 0.369 | 7.75 |
| 76 | 384295 | 398297 | 39.03 | -26.67 | 2.95 | -4.68 | 0.469 | 6.98 | 42.68 | -26.46 | 2.81 | -7.94 | 0.345 | 6.70 |
| 77 | 383277 | 397664 | 40.69 | -25.21 | 3.41 | -5.52 | 0.630 | 4.61 | 43.80 | -25.66 | 3.19 | -7.71 | 0.418 | 5.30 |
| 78 | 382786 | 397728 | 41.31 | -26.02 | 3.01 | -5.00 | 0.574 | 8.28 | 48.39 | -25.85 | 3.91 | -4.62 | 0.492 | 7.21 |
| 79 | 382892 | 397730 | 39.97 | -24.88 | 2.02 | -10.58 | 0.443 | 6.58 | 42.01 | -25.33 | 2.07 | -11.87 | 0.249 | 6.13 |
| 80 | 382661 | 397036 | 40.16 | -25.38 | 3.07 | -5.94 | 0.552 | 8.17 | 48.60 | -25.10 | 3.36 | -6.05 | 0.417 | 7.42 |
| 81 | 384960 | 398477 | 40.04 | -25.39 | 2.68 | -6.34 | 0.478 | 6.82 | 47.56 | -24.78 | 3.31 | -6.51 | 0.399 | 6.46 |
| 82 | 384409 | 397812 | 40.23 | -24.39 | 3.55 | -4.44 | 0.647 | 7.26 | *N/A* | *N/A* | *N/A* | *N/A* | *N/A* | *N/A* |
| 83 | 384382 | 397558 | 39.78 | -25.79 | 3.18 | -7.26 | 0.536 | 7.22 | 47.20 | -25.76 | 3.47 | -8.98 | 0.418 | 7.07 |
| 84 | 382968 | 396568 | 40.09 | -26.38 | 3.28 | -3.84 | 0.551 | 7.38 | *N/A* | *N/A* | *N/A* | *N/A* | *N/A* | *N/A* |
| 85 | 383517 | 398334 | 39.68 | -25.94 | 2.42 | -7.75 | 0.408 | 7.04 | 40.40 | -27.58 | 2.28 | -9.36 | 0.301 | 7.43 |
| 86 | 384256 | 397121 | 39.71 | *N/A* | 3.05 | *N/A* | *N/A* | *N/A* | 45.02 | -25.11 | 3.83 | -7.71 | 0.396 | 6.57 |
| 87 | 383741 | 397851 | 40.43 | -25.39 | 3.36 | -7.34 | 0.655 | 7.59 | 47.78 | -25.81 | 3.60 | -8.53 | 0.450 | 8.14 |
| 88 | 384516 | 398751 | 38.63 | -25.36 | 1.96 | -9.28 | 0.296 | 7.90 | 46.09 | -26.02 | 2.64 | -9.12 | 0.276 | 7.95 |
| Tab. S4 continued: Individual CNS contents (wt%) and stable-isotope ratio signatures (‰), recorded in *X. parietina* (N=94) and *Physcia* spp. (N=86), sampled across Manchester; with XY-coordinates (OSGB1936) and site ID, sampling locations with site-ID shown in Figure S4; N/A – not enough lichen material obtained for analysis | | | | | | | | | | | | | | |
| 89 | 384003 | 398020 | 40.26 | -25.38 | 3.03 | -5.23 | 0.388 | 6.55 | *N/A* | *N/A* | *N/A* | *N/A* | *N/A* | *N/A* |
| 90 | 382488 | 397017 | 40.00 | -24.98 | 2.95 | -4.85 | 0.571 | 9.40 | *N/A* | *N/A* | *N/A* | *N/A* | *N/A* | *N/A* |
| 91 | 382990 | 396950 | 39.39 | -25.05 | 3.41 | -6.87 | 0.602 | 7.39 | 47.18 | -25.73 | 4.19 | -7.08 | 0.443 | 7.59 |
| 92 | 383917 | 397652 | 40.47 | -24.57 | 3.44 | -5.92 | 0.532 | 7.97 | 46.05 | -25.77 | 3.67 | -5.99 | 0.437 | 6.91 |
| 93 | 383349 | 396842 | 36.07 | -24.29 | 2.62 | -7.49 | 0.457 | 7.58 | 40.62 | -25.45 | 3.30 | -7.68 | *N/A* | 7.54 |
| 94 | 385366 | 398773 | 39.23 | -24.31 | 3.10 | -7.49 | 0.630 | 7.38 | *N/A* | *N/A* | *N/A* | *N/A* | *N/A* | *N/A* |


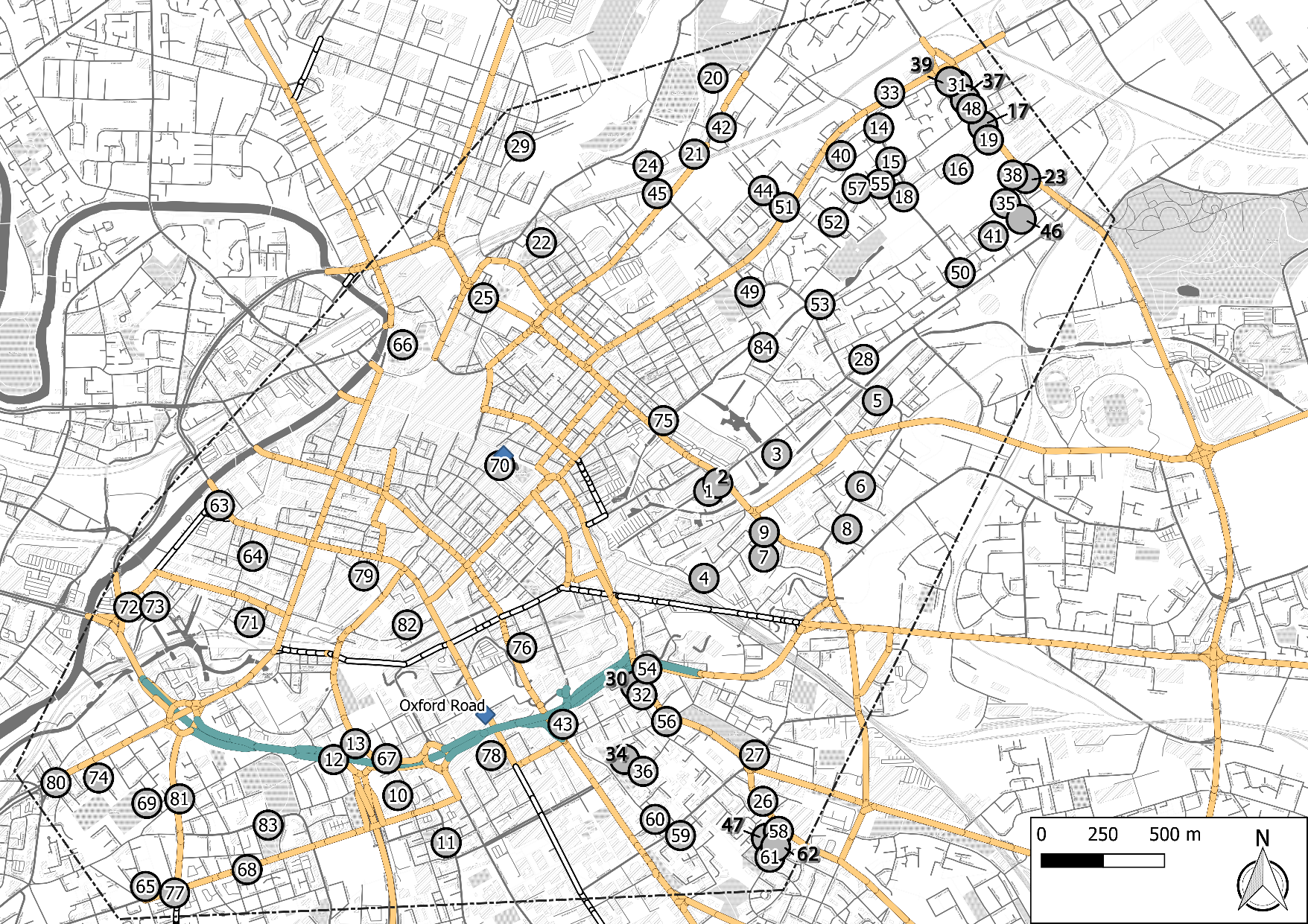


**Fig. S5:** Lichen sampling sites for *X. parietina* (N=94) and *Physcia* spp. (N=86) across Manchester, displayed with Sample-ID and automated monitoring stations (Oxford Road and Piccadilly Gardens; blue rectangles)


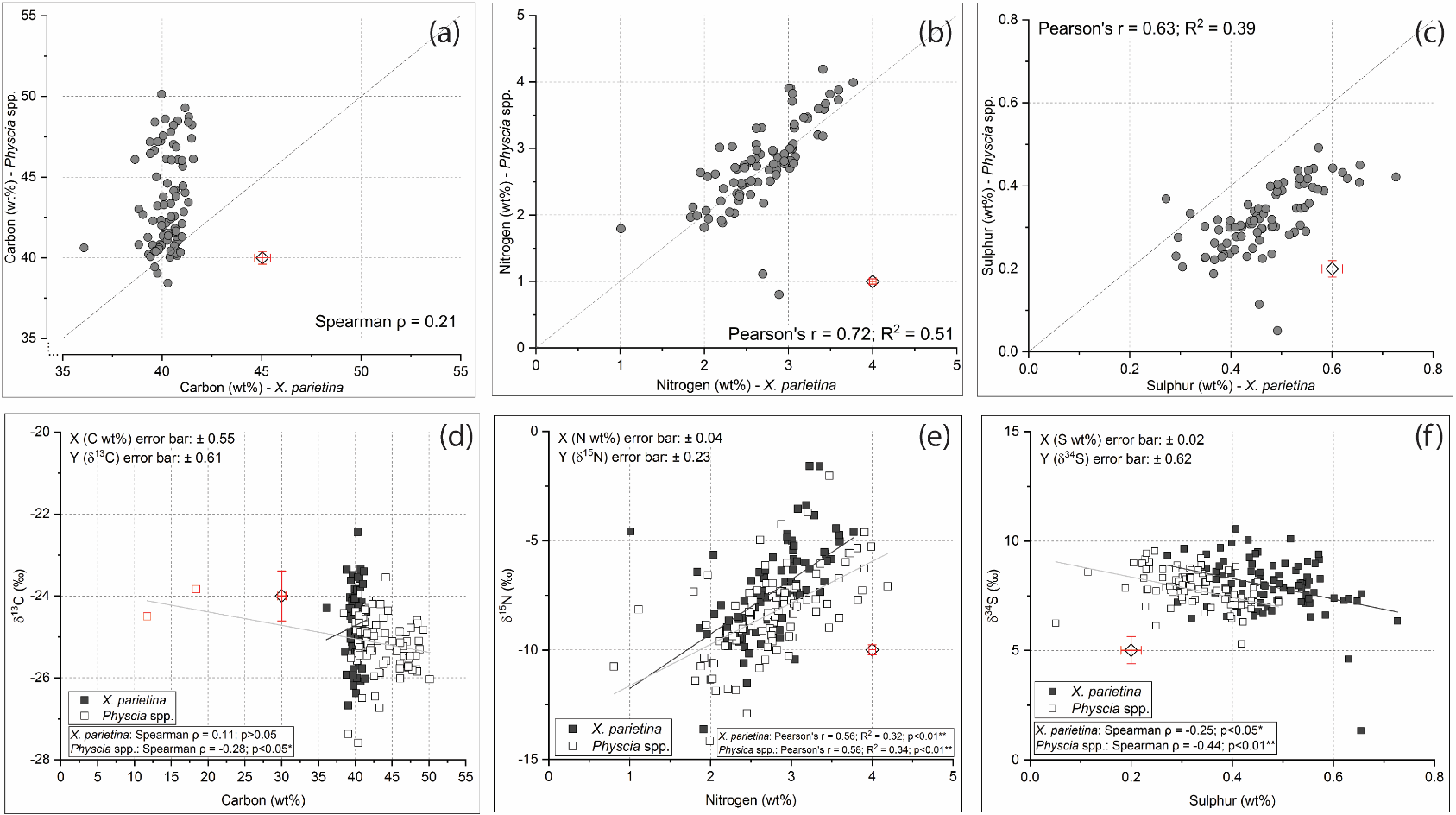


**Fi****g. S6:** Comparison of Manchester urban *X. parietina* and *Physcia* spp. lichens for (a) carbon, (b) nitrogen and (c) sulfur contents, together with scatter-plots of lichen CNS and stable-isotope-ratio signatures (δ^13^C (d), δ^15^N (e) and δ^34^S (f) values), displayed with correlation statistics (Pearson’s r and Spearman ρ and correlation slopes). Analytical errors (± 1x standard deviation) derived from repeated (N=31 for CNS contents; N=43 for stable-isotope ratios) measurements of lichen CRM (No. 482) plotted on dummy data values (white diamond) with wt% C:±0.55; wt% N: ±0.04 and wt% S: ±0.02 and δ^13^C: ±0.61 ; δ^15^N: ±0.23 and δ^34^S: ±0.62; two significant outliers (identified by Grubbs test, p<0.05) in (d) *Physcia* spp. at 11.73 wt% and 18.37 wt% shown as red squares in Fig. S5(d))


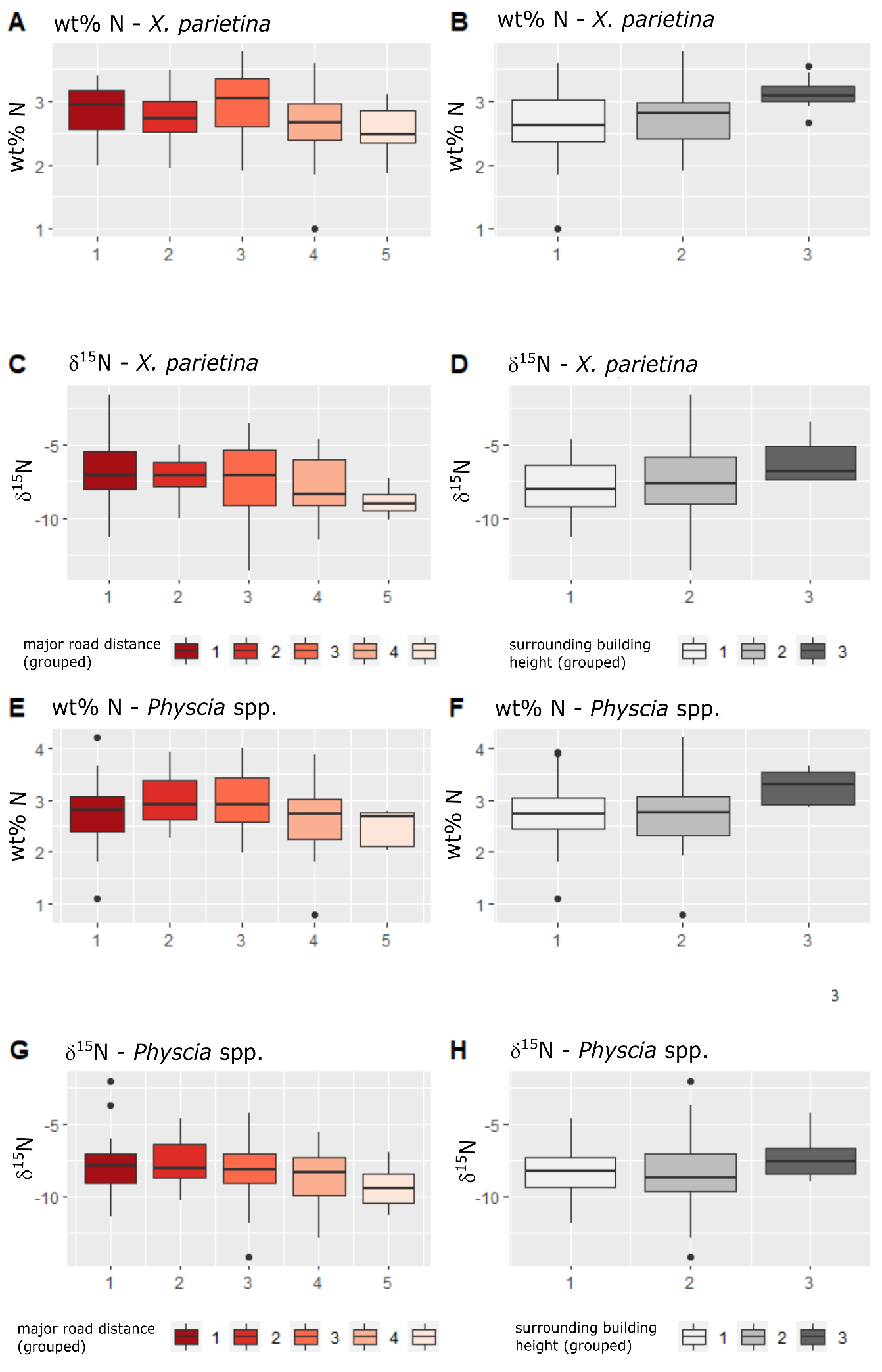


**Fig. S7**: Lichen (*X. parietina* and *Physcia* spp.) wt% N contents and δ^15^N values for grouped urban influencing factors: major road distance (1: <25 m, 2: 25-50 m, 3: 50-100 m, 4: 100-200 m, 5: >200 m) and building heights (1: <10 m, 2: 10-20 m, 3: >20 m)

**Tab. S6:** Correlation matrix of lichen CNS contents (wt%) and stable-isotope ratio signatures (δ^13^C, δ^15^N and δ^34^S) for X. parietina (N=94) and Physcia spp. (N=86) together with NO_x_ diffusion tube measurements for NO_2_ concentrations (N=45 at lichen sampling locations) for a 12-months period (biweekly changes; (Niepsch et al., 2021); Spearman ρ correlation coefficients underlined, other values represent Pearsons’s correlation coefficient; ** significant at the level p<0.01, * significant at the level p<0.05

|  | *Physcia spp.* | C wt% | S wt% | δ^15^N | δ^13^C | δ^34^S | NO_2_ (mean) |
| --- | --- | --- | --- | --- | --- | --- | --- |
| *X. parietina* | **Nwt%** | 0.54** | 0.92** | 0.59** | -0.21 | -0.24* | 0.21 |
| C wt% | -0.08 | **C wt%** | 0.59** | 0.09 | -0.28* | -0.04 | 0.04 |
| S wt% | 0.70** | -0.06 | **S wt%** | 0.51** | -0.19 | -0.44** | 0.28 |
| δ^15^N | 0.56** | -0.10 | 0.51** | **δ^15^N** | -0.10 | -0.21 | 0.37** |
| δ^13^C | -0.23* | 0.11 | -0.16 | -0.32* | **δ^13^C** | 0.21 | -0.40* |
| δ^34^S | -0.27* | 0.19 | -0.25* | 0.37 | ­0.17 | **δ^34^S** | -0.57** |
| NO_2_ (mean) | 0.34* | -0.32 | 0.29 | 0.54** | -0.48* | -0.47* |  |

**Tab. S7:** Carbon, nitrogen and sulphur contents (wt%) and stable-isotope ratio signatures (δ^13^C, δ^15^N and δ^34^S in ‰) of rural *X. parietina* samples; sampled in May 2018 around a poultry farm in Shrewsbury (UK; **Fig. S2**)

| Site-ID | C wt% | N wt% | S wt% | δ^13^C | δ^15^N | δ^34^S |
| --- | --- | --- | --- | --- | --- | --- |
| 1 | 43.3 | 3.44 | 0.353 | -23.4 | -6.36 | 9.44 |
| 2 | 42.8 | 3.34 | 0.332 | -23.0 | -8.09 | 10.9 |
| 3 | 42.0 | 4.22 | 0.420 | -23.1 | 4.44 | 8.42 |
| 4 | 41.5 | 3.81 | 0.521 | -22.8 | 0.03 | 9.96 |
| 5 | 41.4 | 3.95 | 0.515 | -22.0 | -2.69 | 12.8 |
| 6 | 42.2 | 3.87 | 0.453 | -22.3 | -1.05 | 11.0 |
| 7 | 42.8 | 3.51 | 0.427 | -22.8 | -6.59 | 13.4 |
| 8 | 42.7 | 3.21 | 0.403 | -22.6 | -7.61 | 14.2 |
| 9 | 42.0 | 3.39 | 0.235 | -22.4 | -2.67 | 11.0 |
| 10 | 42.2 | 3.42 | 0.400 | -22.1 | -5.95 | 14.1 |
| 11 | 43.0 | 3.09 | 0.342 | -22.5 | -10.1 | 12.7 |
| 12 | 42.6 | 3.40 | 0.462 | -22.6 | -6.76 | 13.5 |


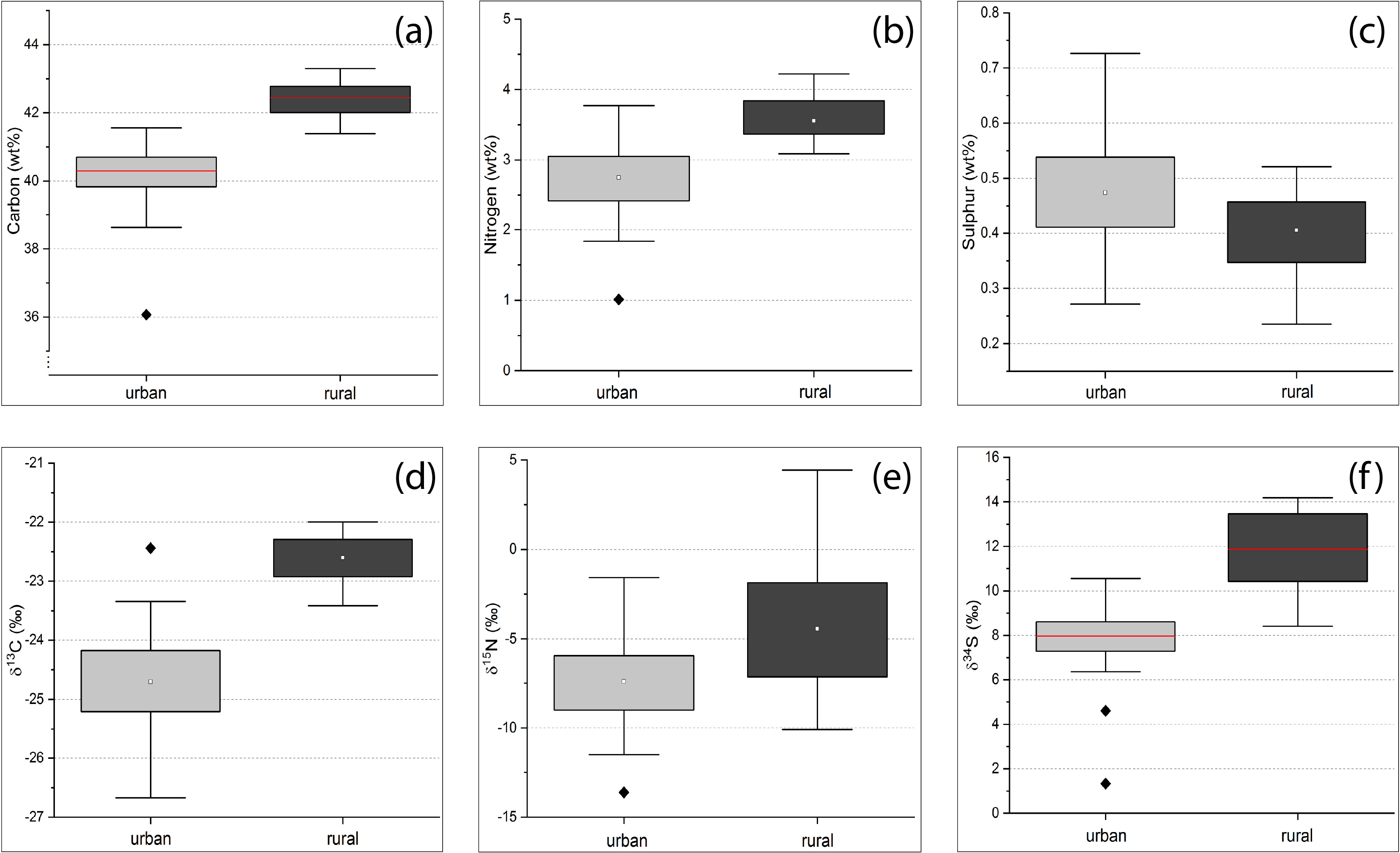


**Fig. S8:** Box-Whisker plots (25th to 75th percentile; displayed with mean: white square for normally distributed data, median line for nonnormally distributed data; extreme values: black diamond) for (a) carbon, (b) nitrogen, (c) sulphur contents (in wt%) and (d) δ13C, (e) δ15N and (f) δ34S values (in ‰) in urban (left) and rural (right) *X. parietina* samples

# **References:**

Air Quality England, 2018a. Air Pollution Report - Manchester Piccadilly (MAN7). Manchester.

Air Quality England, 2018b. Air Pollution Report - Manchester Oxford Road (MAN1). Manchester.

Bermejo-Orduna, R., McBride, J.R., Shiraishi, K., Elustondo, D., Lasheras, E., Santamaría, J.M., 2014. Biomonitoring of traffic-related nitrogen pollution using *Letharia vulpina* (L.) Hue in the Sierra Nevada, California. Sci. Total Environ. 490, 205–212. https://doi.org/10.1016/j.scitotenv.2014.04.119

Britter, R.E., Hanna, S.R., 2003. Flow and Dispersion in Urban Areas. Annu. Rev. Fluid Mech. 35, 469–496. https://doi.org/10.1146/annurev.fluid.35.101101.161147

Browning, M., Lee, K., 2017. Within what distance does “greenness” best predict physical health? A systematic review of articles with gis buffer analyses across the lifespan. Int. J. Environ. Res. Public Health 14, 1–21. https://doi.org/10.3390/ijerph14070675

Buccolieri, R., Sandberg, M., Di Sabatino, S., 2010. City breathability and its link to pollutant concentration distribution within urban-like geometries. Atmos. Environ. https://doi.org/10.1016/j.atmosenv.2010.02.022

Dadvand, P., de Nazelle, A., Figueras, F., Basagaña, X., Su, J., Amoly, E., Jerrett, M., Vrijheid, M., Sunyer, J., Nieuwenhuijsen, M.J., 2012a. Green space, health inequality and pregnancy. Environ. Int. 40, 110–115. https://doi.org/10.1016/j.envint.2011.07.004

Dadvand, P., Sunyer, J., Basagaña, X., Ballester, F., Lertxundi, A., Fernández-Somoano, A., Estarlich, M., García-Esteban, R., Mendez, M.A., Nieuwenhuijsen, M.J., 2012b. Surrounding Greenness and Pregnancy Outcomes in Four Spanish Birth Cohorts. Environ. Health Perspect. 120, 1481–1487. https://doi.org/10.1289/ehp.1205244

DfT, 2017. Road traffic statistics [WWW Document]. URL https://www.gov.uk/government/publications/road-traffic-estimates-great-britain-jan-to-mar-q1-2014%5Cnhttps://www.gov.uk/government/collections/road-traffic-statistics

Digimap - Ordnance Survey, 2018. OS Open Greenspace [WWW Document]. URL https://digimap.edina.ac.uk/webhelp/os/data_information/os_products/os_open_greenspace.htm (accessed 12.12.18).

Digimap - Ordnance Survey, 2017. OS Building Heights (Alpha) [WWW Document]. URL https://digimap.edina.ac.uk/webhelp/os/data_information/os_products/os_building_heights.htm (accessed 12.11.18).

Digimap - Ordnance Survey, 2016. OS Open Roads [WWW Document]. URL https://www.ordnancesurvey.co.uk/business-and-government/products/os-open-roads.html (accessed 6.17.17).

Gombert, S., Asta, J., Seaward, M.R.., 2003. Correlation between the nitrogen concentration of two epiphytic lichens and the traffic density in an urban area. Environ. Pollut. 123, 281–290. https://doi.org/10.1016/S0269-7491(02)00367-6

Hertel, O., Goodsite, M.E., 2009. Urban Air Pollution Climates throughout the World. Air Qual. Urban Environ. 1–22. https://doi.org/10.1039/9781847559654-00001

Janhäll, S., 2015. Review on urban vegetation and particle air pollution – Deposition and dispersion. Atmos. Environ. 105, 130–137. https://doi.org/10.1016/j.atmosenv.2015.01.052

Laffray, X., Rose, C., Garrec, J.P., 2010. Biomonitoring of traffic-related nitrogen oxides in the Maurienne valley (Savoie, France), using purple moor grass growth parameters and leaf ^15^N/^14^N ratio. Environ. Pollut. 158, 1652–1660. https://doi.org/10.1016/j.envpol.2009.12.005

Lo, K.W., Ngan, K., 2015. Characterising the pollutant ventilation characteristics of street canyons using the tracer age and age spectrum. Atmos. Environ. 122, 611–621. https://doi.org/10.1016/j.atmosenv.2015.10.023

Niepsch, D., Clarke, L.J., Tzoulas, K., Cavan, G., 2021. Spatiotemporal variability of nitrogen dioxide (NO_2_) pollution in Manchester (UK) city centre (2017–2018) using a fine spatial scale single-NOx diffusion tube network. Environ. Geochem. Health. https://doi.org/10.1007/s10653-021-01149-w

Quevauviller, P., Herzig, R., Muntau, H., 1996. The certification of the contents (mass fractions) of Al, As, Cd, Cr, Cu, Hg, Ni, Pb and Zn in lichen CRM 482.

Salmond, J.A., Williams, D.E., Laing, G., Kingham, S., Dirks, K., Longley, I., Henshaw, G.S., 2013. The influence of vegetation on the horizontal and vertical distribution of pollutants in a street canyon. Sci. Total Environ. 443, 287–298. https://doi.org/10.1016/j.scitotenv.2012.10.101

Shen, J., Gao, Z., Ding, W., Yu, Y., 2017. An investigation on the effect of street morphology to ambient air quality using six real-world cases. Atmos. Environ. 164, 85–101. https://doi.org/10.1016/j.atmosenv.2017.05.047

Whitworth Meterological Observatory - Data Archive [WWW Document], 2018. URL http://whitworth.cas.manchester.ac.uk/2018/ (accessed 10.16.18).
